# COVID vaccine stigma: detecting stigma across social media platforms with computational model based on deep learning

**DOI:** 10.1007/s10489-022-04311-8

**Published:** 2022-12-07

**Authors:** Nadiya Straton

**Affiliations:** Department of Digitalisation, Copenhagen Business School, Howitzvej 60, Frederiksberg, 2000 Denmark

**Keywords:** COVID-19, Vaccine, Stigma, Deep learning, Social media

## Abstract

The study presents the first computational model of COVID vaccine stigma that can identify stigmatised sentiment with a high level of accuracy and generalises well across a number of social media platforms. The aim of the study is to understand the lexical features that are prevalent in COVID vaccine discourse and disputes between anti-vaccine and pro-vaccine groups. This should provide better insight for healthcare authorities, enabling them to better navigate those discussions. The study collected posts and their comments related to COVID vaccine sentiment in English, from Reddit, Twitter, and YouTube, for the period from April 2020 to March 2021. The labels used in the model, “stigma”, “not stigma”, and “undefined”, were collected from a smaller Facebook (Meta) dataset and successfully propagated into a larger dataset from Reddit, Twitter, and YouTube. The success of the propagation task and consequent classification is a result of state-of-the-art annotation scheme and annotated dataset. Deep learning and pre-trained word vector embedding significantly outperformed traditional algorithms, according to two-tailed P(T≤t) test and achieved F1 score of 0.794 on the classification task with three classes. Stigmatised text in COVID anti-vaccine discourse is characterised by high levels of subjectivity, negative sentiment, anxiety, anger, risk, and healthcare references. After the first half of 2020, anti-vaccination stigma sentiment appears often in comments to posts attempting to disprove COVID vaccine conspiracy theories. This is inconsonant with previous research findings, where anti-vaccine people stayed primarily within their own in-group discussions. This shift in the behaviour of the anti-vaccine movement from affirming climates to ones with opposing opinions will be discussed and elaborated further in the study.

## Introduction

Very often, vaccination discussions use language projections that transform into strong stigmatised opinions against groups who are involved in healthcare, government institutions or individuals who choose to vaccinate and vice versa with pro-vaccination groups against individuals who do not want to vaccinate. On the other hand such stigmatised sentiments perpetuate antagonism and hostility between pro-and anti-vaccine groups. On the other hand, they reinforce fear and doubt about vaccines’ side effects, leading to disputes about effectiveness overall. COVID vaccines are probably not unique in that respect; however, they began attracting negative, discrediting comments long before they were developed, which is probably unparalleled and also a very dangerous development for the pandemic’s course. These discrediting comments can be explained by the unconscious tendency to assign more blame and stigma to conditions that seem more threatening and unknown than conditions that are perhaps equally dangerous but are better understood, as was observed in [1] and [2].

This study’s results can help to design a model for identifying stigmatised sentiment in discussions about vaccines, both those developed during the ongoing pandemic and those that have been on the market for many years but still face resistance. Building a computational model of COVID vaccine sentiment is not a trivial task. It requires defining the concept of stigma and then, translating it into a computational model that can identify such sentiment in a text. As for the definition of *stigma*, etymologically, it comes from the Latin stigmat, meaning “mark” or “brand”, from the Greek stizein, meaning “to tattoo” and was first mentioned in English texts in reference to a “scar from an iron”. However, in modern use, “scar” or “mark” is used in a metaphorical sense to represent a set of negative, often unfair beliefs, and a mark of shame projected by one person or a group of people unto another person or group [3].

*Stigma* denotes an unusual and negative thing about a signifier that must bear the mark of discredit to identify an abomination of the body, blemishes of an individual character, or a tribal/group taboo [4]. *Stereotype* represents an oversimplified opinion, also described as the primary rationalisation of displaced frustration [5]. *Bias* is a personal unreasoned judgement, while *prejudice* is an irreversible prejudgement directed against a group for their supposed characteristics, expressed through projection, animosity, anxiety, and dichotomisation among others [5]. This study used these concepts interchangeably since they share a similar sentiment and can quickly lead to discrimination given the right conditions [5].

Most stigmas, prejudices, and stereotypes have an inherent element of threat and are characterised by ambivalence and contradictory ideas about someone or something [1]. These opposing ideas may represent two aspects about out-group members, for example “incompetent but warm” and “competent but not warm” [6]. The out-group can attract subjectively positive feelings that coexist with feelings of antipathy [7]. Members who exhibit both aspects of positive sentiment competent and warm are in-group members, resulting in in-group favouritism [6].

Therefore, it is often very difficult for in-group members of anti-vaccination or pro-vaccination groups to acknowledge anything positive about the out-of-group. Vaccine communities tend to favour information that reinforces their preconceived view, according to the selective exposure [8, 9] confirmation bias theories [10–12]. The confirmation bias pervasive in those discussions is likely to endorse a hypothesis that conforms to the in-group belief rather than the truth and therefore expressing the truth might mean betraying one’s own community [10, 12]. People strive for internal psychological consistency to mentally function in the real world, so that people who experience internal inconsistency tend to be psychologically uncomfortable and motivated to reduce cognitive dissonance [13]. Some are so uncomfortable and stressed by such polarised ideas that they resolve the situation by blindly defending the point of view that they want to support.

Leon Festinger argued that this especially happens in perturbed situations when disagreement becomes more intense despite all parties being exposed to the same evidence [13]. People also justify their behaviour by rationalisation or avoiding circumstances where they can be confronted with contradictory information or opposing opinions. Comments that members deem offensive and conflicting with the in-group view can result in their deletion or blocking of the contributor to the page [14]. Such anxiety during any interaction with out-group members can be caused by stereotyping, dissimilarity, and lack of contact (keeping only to in-group conversations) [15].

“Anti-vaxxer” accounts on social media sites like Facebook (Meta), Twitter, YouTube, and Instagram reach more than 59 million followers [16]. The 12 biggest accounts are responsible for 65% of the alleged disinformation shared online [17] and spread over a dozen platforms [18], which primarily concerns vaccines developed in Western countries. Some influencers have been offered money to spread misinformation [18]. Additionally, a media company was used as a platform for spreading alarmist headlines about Pfizer vaccine side effects and theories that the public inoculations of politicians are a hoax [19]. Claims that infertility is a side effect from the Pfizer vaccine have been circulating on YouTube since early 2020 and are complicated by the absence of data on the impact of vaccines on pregnant women [20]. High demand for and a low supply of information create an uncertainty vacuum in which conspiracy theories and prejudiced views flourish [5, 20].

Johnson et al. suggested that the growth rate of an influential anti-vaccination movement can be curbed if they are intervened with, although the outcomes of intervention had not been researched [21]. In 2019, Facebook (Meta) started removing posts about vaccine hoaxes, across the platform, including in private pages and groups [16]. There were also attempts in mid-late 2020 to ban the most prolific anti-vaccination accounts [22]. In February 2021, Facebook (Meta) widened its ban on vaccine misinformation and pledged to remove claims that vaccines are not effective against diseases, vaccines cause autism, that it is safer to contract COVID-19 than to receive the vaccine and so forth, in effect removing around 2 million pieces of widely debunked content [16, 23].

Despite these limitations, anti-vaccination accounts partially bounce back by moving to different platforms or joining forces with other groups, such as anti-government groups [22], given that the main scapegoats for anti-vaccination communities are government institutions, pharmaceutical companies, and health authorities [24]. Some anti-vaccination contributors get around moderation policies by posting through so-called echo chambers or filter bubbles in comment sections of the news on Facebook (Meta) as they are not subjected to warning labels by third-party fact-checking partners [25].

One of the findings of the current research is that prejudiced sentiment and conspiracy theories about COVID vaccines have been circulating in the comment sections of health authorities that try to disprove COVID-19 conspiracy theories. The latter suggests that efforts to curb anti-vaccination pages have a counter-productive effect and might not be the best strategy for dealing with the anti-vaccination movement. Moreover, it can be seen through observation that anti-vaccine pages on Facebook (Meta) started to form even more tightly-knit, exclusive communities with accounts set to private view. The following research questions aimed to shed light on COVID vaccine stigma and its features through anti-vaccine and pro-vaccine discussions on social media domains:

1. How can rigorous computational model identify COVID vaccine stigma across social media platforms?
2. Is there a significant computational advantage among the models for identifying COVID stigma in the study?
3. Which textual features are characteristic of COVID vaccine discourse stigma and which features are preferable in communication on the topic?
4. Does the COVID vaccine stigma lead to disengagement with content or is the reverse true?
5. How can the stigma and friction in vaccination discourse be reduced on social media platforms? Why might that be important?

The first research question is addressed in Section 3, Materials and Methods; Section 4, Results; and Section 5, Discussion and Conclusion. The second research question is addressed in the Section 4.3, Classification models; and is concluded in Section 5. The third research question is answered in Section 4.4, Features; and in Section 5. The forth research question is discussed in Section 4.4 and Section 5. The fifth research question is put forward in the Introduction and addressed in Section 5, Discussion and Conclusion.

## Literature background

The body of literature was searched for healthcare stigma research conducted on social media sites and online forums from 2015 to 2020. Research conducted by the author was excluded from the initial review. Additional studies were added based on the the key words “COVID stigma”, “COVID vaccines” for the period 2020 to 2021. After several screening rounds, out of an initial 5209 studies, 12 studies were included in the final selection, based on their quality and relevance. An additional four studies that discussed COVID vaccines, were also incorporated. The primary focus of current research is to explore studies that either try to identify stigma in social media posts or study stigma from social media content that was directed at certain preventive measures or health-related issues.

Five quantitative and seven qualitative/mixed studies were identified from the initially reviewed articles. Among these, 67% of studies [26–28, 31, 32, 35, 36] examined various mental health stigma, [29] analysed suicide stigma, [30] talked about vaccine stigma among mothers, who refuse vaccines for their kids, [37] discussed stigma linked to COVID pandemic, and [33, 34] explored weight stigma. The additional four studies about COVID vaccines are primarily theoretical articles. [38, 39, 41] discussed pro-and anti-vaccine attitudes. [40] used mixed approach and discussed polarisation of attitudes towards the COVID vaccine based on political affiliation.

Machine learning techniques were applied primarily in quantitative research to build classification models [27–29, 32, 36]. An F1 score of 72.79% was achieved using a Decision Tree technique to classify stigmatising vs. non-stigmatising sentiment and Cohen’s k of 0.73 inter-rater agreement [29]. Similarly, [27] obtained F1 of 75.20% using Random Forest model. Comparable or higher F1 measure was achieved in the present study using CNN.

In [32], two researchers manually coded 311 randomly selected tweets and assigned six dimensions with varying degrees of inter-rater agreement. “Metaphorical”, “organisation”, “informative”, “personal”, and “joke” were linked to stigma in [32]. Joke, organisation, informative, figure of speech do not always infer stigma. In [36] content analyses of tweets was conducted by one of the authors, where colloquialism was concluded to represent stigma. In such cases, quantitative models will look for colloquialism, metaphor, and so on rather than stigma sentiment. Stigma can be expressed in various linguistic styles, however it does not mean that metaphor or colloquialism should be presented as stigma.

Qualitative or mixed studies also use misnomers in the definition of stigma [30, 31, 35]. Although there are exceptions in [26] and [34]. “Social distance”, “mocking/trivialising”, “self-stigma”, “inaccurate beliefs”, “dangerousness”, and “negative sentiment” illustrate the concept in [26]. A “Fat” stigma was deemed to be any form of devaluation in [34], such as teasing, bullying, ridicule, and physical violence. “Gluttonous”, “lazy”, “stupid” key words are correlated with the domain of weight stigmatisation [33]. Similarly key words “Chinese virus” or “China virus”, pre-determine a tweet to be stigmatised within the domain of COVID pandemic [37]. Such key words can hardly be propagated to study stigma in other domains. Most studies in the review [26–29, 31–36] used one or two authors/researchers to annotate data or derive classes based on key topics.

While authors of the article acting as data annotators might lead to better inter-rater agreement, it might also introduce the author’s bias into the model, where annotated data has direct influence on the model’s outcome. Moreover, according to good annotation practice, measuring inter-rater reliability based on two annotations or assignments per post is rarely considered enough [49]. Each post/comment in the current research was classified on categories of stigma, not stigma, or undefined. The “gray zone” of the undefined category and its features had not been previously studied and is of interest to the current research. Because, the data has three independent expert annotations per post, with the fourth assignment in cases of disagreement, the annotated data can be considered reliable.

The Annotation in the current research is not limited to healthcare context or vaccination discourse and can be applied to studying the concept of stigma across a wide variety of disciplines. A majority (37%) of the studies [26, 31–33, 36, 37] were based on Twitter data. While [27–29] derived data from SinaWeibo and [30, 39] studied interview questionnaires. [40] discussed stigma based on Facebook data, [35] analysed data from online forum, and [34] explored stigma on YouTube. Both [41] and [38] mentioned various social media sites in the discussion.

To the best of the author’s knowledge, the current study introduced the only computational model that can identify COVID vaccine stigma across several social media domains (Facebook, Twitter, YouTube, and Reddit). The differences between those social media domains are substantial in terms of the length of the text, engagement parameters, users, and the way information is communicated and therefore they serve as a good test for the model’s performance. The current study fills the gap of reliable, rigorous annotation process and scheme that reflects main research works on stigma and can be applied in other domains beyond the vaccination discourse. Moreover, the study attained good classification result with pre-trained deep learning models together with some traditional models. Models were selected based on the problem description and type of the data with unbalanced classes.

## Materials and methods

### Study design

The main purpose of this study was to build a model that can identify COVID vaccine stigma with high levels of precision and then analyse its outcomes. The present study used a cross-sectional approach, because it was more important to identify the stigma features and differences between stigmatised sentiment and non-stigmatised sentiment in a given period of time rather than to study changes of the concept over time with a longitudinal approach.

The development of an annotation scheme and process necessitated the inclusion of elements of an experimental nature. The initial nine short annotation categories were updated to become state-of-the-art, with fewer categories. This was also a result of continuous feedback from trained annotators and estimates from Cohen’s and Fleiss’s kappa rates of agreement. The main body of work is non-experimental quantitative and the source data are not tampered with, due to the identification of stigma and its characteristic features being central to the study. This study used an analytic observational and retrospective approach, with elements of a quasi-experiment. Moreover, the study did not collect any sensitive information; therefore, no special permission was necessary to process the data. The data were shared by private individuals on social media pages consensually and publicly.

### Data collection

COVID vaccine data were collected in English from social media domains (Reddit, Twitter, and YouTube) retrospectively for the period from April 2020 to March 2021. This includes the time before the COVID vaccine rollout and go through roughly 3 months after the first person was vaccinated with the Pfizer vaccine on the 8 of December 2020 [42]. The collection of data included posts with stigmatised sentiment towards COVID vaccines and the comments, as well as posts that sought to disprove the COVID vaccine conspiracy and its comments.

Reddit posts and comments were collected through PRAW using python script, Twitter data were collected with Octoparse [43], and YouTube data were collected with YouTube Comment Scrapper [44]. To collect data from Reddit, the search phrase “COVID vaccine” was used to compile posts in Conspiracy subReddit. Posts with stigmatised sentiment were selected according to the criteria presented in the stigma annotation scheme shown in Table 1. Criteria for the collection of the content were posts that correspond to the definition of stigma presented in the annotation scheme and corresponding to the minimum of three components of the definition. The latter included but is not limited to blame, conflict (hate, fear), suspicion, rejection, inflexible unfounded overgeneralisation, one-sided interpretation, and dichotomisation.

**Table 1:** Annotation scheme - 4 labels

Does the sentiment convey stereotype/prejudice/bias/stigma? If YES 1-2, if lacking context - 3, if NO then 4 (NONE)		
Label	Post/Comment example	Literature references that infer annotation labels
1. Expressions that sustain hostility: (i) Blame, Suspicion (ii) Conflict (Hate, Fear) (iii) Exaggeration, Strong emotion, An insult, Rejection, Animosity, Condescension, Aggression	i) “So today I heard that if you don’t have covid and you get tested, they literally put the virus on the swab they test you with to infect you. the goal is to have everyone positive so we’re forced to get the covid-19 vaccine (which will have a microchip in it).” ii) “The COVID apartheid is gathering pace Spain intends to set up a registry of people refusing a vaccine. This would be shared with other EU countries. This infringement of civil liberties sets a dangerous precedent. Freedoms lost are rarely regained easily.” iii) “Bill Gates says Trump claim about COVID cure is ‘inappropriate’ Oh so ‘doctor’ Gates wouldn’t be able to sell his dodgy vaccine if Trump’s drug works... ”.	i) Ad-hoc scapegoats might not be lily-white, but they always attract more blame [5]. Frustration generates aggression, which becomes displaced on relatively defenceless goats, is rationalized by blaming, projecting, stereotyping [5]. Suspicion of the out-of-group comes from fear of defeat or by default [45]. Most stigmas hold an element of threat [1]. ii) Evidence about subtleness of stigma suggest that fear, may be part of the sentiment [46]. Externalization of conflict (it is not I who hates and injures others, it is they who hate and injure me) [5]. iii) E. Goffman outlines that one way to express stigma is to point to blemishes of individual character such as weak will, domineering nature, dishonesty, wrong political views etc. [4]. Anger is an emotion directed at a single object, hatred is a sentiment directed at the whole class [5]. Under certain circumstances there will be step-wise progression from verbal rejection to violence [5].
2. Expressions that sustain inconsistency and over-generalization: (i) Inflexible unfounded overgeneralisation, One−sided interpretation (ii) Predicting, guessing (iii) Unsupported judgement, Personal opinion, Projection (iv) Dichotomization, Tabloid thinking, Demagoguery	i) “...Test and Trace - dead Lockdowns - exposed as ineffective Curfews - useless Mass Testing - full of inaccuracies Covid deaths - questionable data Vaccine - rushed and suspect nothing this government and SAGE do has any credibility.” ii) “..I can guarantee the brainwashed will be flocking to get the jab! They’ve probably not made as much money on the flu jabs this year.. Scaremongering!” iii) “So what happens when everyone who gets the vaccine then tests “positive” for “Covid”? I know! The government continues to lock us down, destroys our lives and livelihoods. Oh, plus a “new strain”. Rinse repeat until all small business is destroyed and we’re all desperate/destitute”. iv) “32.7m people have died of HIV/AIDS in the last 35 years 690,000 died in 2019 alone. There is no vaccine for HIV/AIDS despite best efforts over those 35 years COVID though? 6 months and 3 companies have a vaccine which is 90% effective. Sound plausible?”	i) If the people being judged are outgroup members, the perceiver will see them as especially similar, lacking in variability [47]. ii) Uncertainty fuels prejudice [5]. There is interest in imaginative processes, in fantasies, in theoretical reflections, in artistic activities [5]. iii) Based on the input from annotators: “personal opinions and projections which are not substantiated feel like stigma/prejudice”. Favorableness or unfavorableness that accompanies unsupported judgement and is not based on previous experience [5]. iv) Prejudiced person is given two valued judgement and dichotomizes when things of nature, of law, of morals [5]. Demagoguery justifies and encourages tabloid thinking, stereotyping, and conviction that the world is made up of swindlers [5].
3. Lacking context to make a decision	“I’m a little confused. I thought Kennedy wasn’t for forced vaccinations.” “I can’t even breathe w one freaking mask. Ridiculous.” “Then you should have no worries volunteering yourself ... take the trial vaccines as you know so much about vaccines abi?”	
4. Not stigma	“Please Sir, what other option do you have aside vaccine?” “Herd immunity for thee, vaccine for meeeee.” “Heard from who? Sources? Proof?”	

For Twitter, the same search criteria were applied along with the condition that the posts should have accrued a minimum of 50 comments, 50 retweets, 50 likes, and sorted by the “top” posts. The most prevalent topics in COVID vaccine debates on YouTube were about conspiracy and side effects. Therefore, data were collected from YouTube videos with a minimum of 50 comments using the search phrases “COVID vaccine conspiracy” and “COVID vaccine side effects/serious side effects”.

### Data model and analyses

According to Kang-Xing Jin, head of health at Facebook (Meta), despite all of their screening efforts, vaccine comments are “nuanced”, which makes it difficult to discern between people’s personal experiences of feeling sick after being vaccinated and content aimed at discrediting and misinforming [23]. Similar challenges were faced in the current study because the main purpose was to discern stigmatised discrediting posts from personal experiences to understand the reasons for polarisation in the vaccination debate, engagement with stigmatised content, and possible ways to narrow the gap of contrariety of opinion between anti-vaccine and pro-vaccine groups. In order to build a model that identifies stigmatised sentiment, the concept must first be defined.

However, in addition to the lack of general consensus among researchers on the definition of the concept, stigma sentiment is multifaceted and thus requires rigor in designing an annotation scheme with definitions and an annotation process. Link and Phelan (2000) pointed out, “The stigma concept has been applied to an enormous array of circumstances. Each one of these is unique and each one is likely to lead investigators to conceptualise stigma in a somewhat different way” [46]. Link and Phelan (2000) elaborated that the concept is multidisciplinary, with contributions from various disciplines. Even within a single discipline, researchers approach the concept from various theoretical angles, which also leads to different interpretations [46].

One of the challenges mentioned by authors in relation to the concept is that interpretation by social science researchers is from the theoretical perspective rather than lived experience [46]. Taking into account the complexity of the concept, during the annotation process, the present study arrived at a construct that includes those characteristics most established by the research community along with feedback from laymen. The convoluted concept based on theoretical frameworks from [1, 4, 5, 45–47] was split into simpler definitions centered around characteristics, which are presented in Table 1.

Most labels stem from the literature; however, the category “personal opinion/projection” was derived through an annotation process and might reflect the lived experiences of the annotators.

Initially, the annotation schema contained nine categories, but it was later clustered into four groups: hostility stigma, overgeneralisation stigma, undefined, and not stigma. The hostility stigma represents a stronger stigma sentiment than the inconsistency/overgeneralisation stigma and was easier to identify in the texts, which is reflected in the better annotation agreement rate for the category. The annotation schema evolved from the process described in [48] to the schema shown in Table 1 with literature definitions and post/comment examples. The comments originate from the YouTube, Reddit, Twitter COVID stigma dataset described in Table 2. The literature references reveal label definitions and clarify reasons for the selection. Each comment was annotated three times, except that a fourth annotation was conducted in the event of lack of consensus on the category assignment. Comments referred to as “markable” were annotated by a set of annotators (*c*), who assigned labels from a set of categories (*k*) presented in the annotation schema. Observed agreement (*Ao*) measured the percentage of judgements on which the annotators agreed when independently coding the same data (divided by the total number of data points) [49]:

$$ Ao =\frac{1}{i} \sum\limits_{i\in I}{arg_{i}},  \arg_{i} \text{for all items } i \in I $$

where:

$$ \begin{array}{@{}rcl@{}} \arg_{i} = \begin{cases} 1 & \text{if the three coders assign i to the same category}\\ 0 & \text{if the three coders assign i to different categories}\\ \end{cases} \end{array} $$

**Table 2:** Datasets featuring posts and their commments in COVID vaccination discourse

Dataset description			Sentiment						Engagement value		
	# Posts	# Comments	# Stigma	# Not stigma	# Undefined	% Stigma	% Not Stigma	% Undefined	Min	Max	Average
COVID vaccine stigma (posts)											
Reddit	15	4814	1204	3439	186	24.93	71.22	3.85	− 62	16624	14
Twitter	26	2252	451	1676	151	19.80	73.57	6.63	0	194305	141
YouTube	6	1788	367	1291	136	20.46	71.96	7.58	− 11	17869	24
Total	47	8854	2022	6406	473	22.72	71.97	5.31	− 62	194305	49
Disproving COVID vaccine stigma (posts)											
YouTube	59	31230	6692	23106	1491	21.39	73.85	4.77	− 757	1515570	35

Eleven annotators were recruited through a personal network. All of them had some social science background. They were of various ages and both genders were represented. Roughly the same number of annotators were recruited through Amazon MTurk. The annotators independently assigned three labels to each comment. Fleiss kappa was an appropriate measure for quantifying the chance agreement that reflects the combined judgements of all of the coders [50]:

$$ P(k ) = \frac{1}{ic} n_{k} $$

where *P*(*k*) is the expected agreement, *i* is the total number of assignments, *c* is the number of coders, *n*_*k*_ is the number of times an item *i* was classified in category *k*. Fleiss Kappa of 0.84 (*P*(*k*)), 89% share of agreement was attained with two annotated labels: “stigma” and “not stigma”. Fleiss Kappa of 0.62 (*P*(*k*)) and share of agreement 68% was achieved with three classes: “stigma”, “not stigma”, and “undefined”. However, the present study was based on three classes because the gray zone of the undefined class is of interest in terms of its features.

**Fig. 1:**
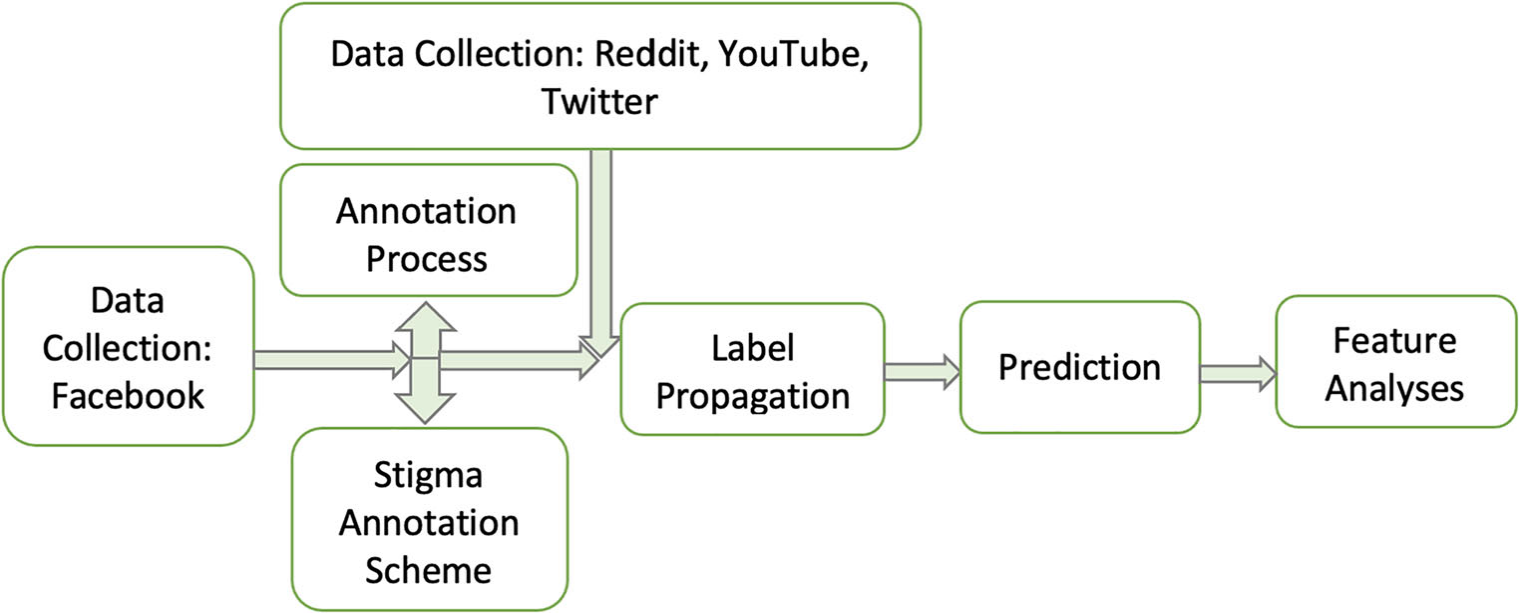
Data model process

The process continued with label propagation and the consequent COVID vaccine stigma feature analyses, as shown in Fig. 1. The data model in Fig. 1 is the process that began with the initial data collection from Facebook (Meta) and the annotation of every post/comment by three annotators, followed by propagating the labels to a larger dataset from Reddit, YouTube, and Twitter. Machine learning models were applied on the propagated dataset to evaluate the traditional model’s performance against deep learning models, such as logistic regression, random forest and pre-trained CNN with Glove, FastText, ELMo, and Gensim embeddings. Eventually, features were analysed for each of the stigma, not stigma, and undefined labels, with linguistic and psychological categories from Linguistic Inquiry and Word Count (LIWC) [51]. Additional features in the model include scores for sentiment polarity, subjectivity, and engagement.

Analysis of variance (ANOVA) F value was used to determine if the continuous variables/features were significant for the classification task (i.e how well they discriminate between multiple classes). The 30 best features were selected with SelectKBest in scikit-learn. The ANOVA F value formula is as follows *F* − *value* Anova Formula [52]:

$$ F = \left( \frac{SSE1-SSE2}{m}\right) / \frac{SSE2}{n-k} $$

where SSE is the residual sum of squares, m is the number of restrictions, and k is the number of independent variables. The backward selection of predictors through recursive feature elimination (RFE) is another way to establish the *n* most important features through elimination. It was implemented with scikit-learn using the RFE algorithm implementation presented in [53]. The *z*-score calculates how many standard deviations above or below the population mean a data point (feature) is. *z*-score Formula:

$$ z=\frac{{y-}{\overline{y}}}{s}=\frac{x-\mu}{\sigma}=\frac{DataPoint-Mean}{StandardDeviation} $$

The emotional tone feature of stigmatised sentiment deviates from the general emotional tone for the total population according to the data displayed in Table 4 and Section 4, stigmatised sentiment is expressed in less emotion (a negative *z*-score value of − 5.9594).

## Results

The dataset displayed in Table 2 has 40,190 posts/comments, where 8,714 (21.88%) show stigmatised sentiment, 29,512 (73.43%) show not stigmatised sentiment, and 1,964 (4.88%) exhibit undefined sentiment. Undefined sentiment means anything difficult to construe and assign to either category.

**Fig. 2:**
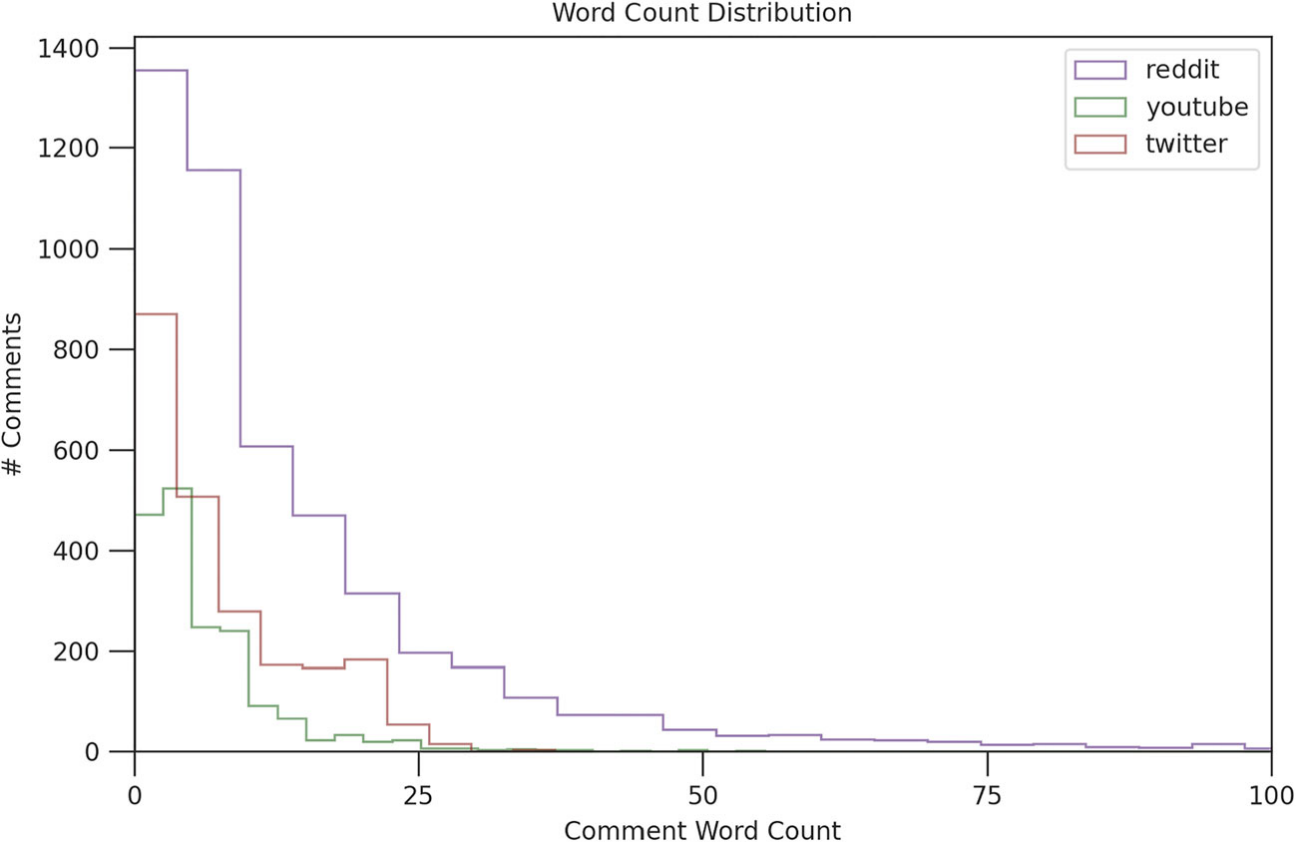
COVID Vaccine Stigma Dataset (Reddit, Twitter, YouTube) with word count per comment

Engagement values are based on Likes (Twitter), Retweets (Twitter) and Upvotes/Downvotes (Reddit, YouTube), with the latter showing both negative and positive engagement. On average, stigmatised posts from Reddit attracted more comments and were also more extensive than comments on Twitter and YouTube, as seen in Fig. 2. The relatively shorter length of comments on Twitter is due to the limit of 280 characters [54], with only 12% of comments being longer than 140 characters. However, the character limit on YouTube is set to 10,000, so it is perplexing why comments are so brief on this site [55]. The number of tweets is limited to 2,400 per day and comments to YouTube posts are limited to 500 [56].

Several different types of stigma, such as subtle generalisations and expressions that sustain hostility, are likely to be included in one long comment to a stigmatised post on Reddit, which is different from comments on Twitter or YouTube. This could further explain why a higher proportion of stigma sentiment was discovered on the Reddit platform in comparison to the other two platforms (the findings also suggest that stigmatised sentences tend to have more characters). The COVID stigma sentiment was identified through label propagation from a smaller annotated dataset of vaccination discourse on Facebook (Meta), which comprises 2,761 comments containing anti-vaccination and pro-vaccination sentiment and about 60% of comments showing stigma/prejudice/stereotype. The process is described in greater detail in [48] and [24].

### Dataset

All of the posts/comments in the current study (Table 2) were collected around June and July 2020, after attempts were made by social media companies to close anti-vaccination accounts. However, some anti-vaccination pages are still in existence. In particular, a search of Facebook (Meta) using the keywords “vax” and “vaccine/s” returned 117 accounts primarily discussing vaccines prior to the start of the COVID-19 pandemic in February 2019.

Out of 117 accounts, 60.68% were anti-vaccine pages (no. followers: avg. = 17,947, min. = 54, max. = 210,307) and 39.32% were pro-vaccine (no. followers: avg. = 25,003, min. = 68, max. = 226,242). At the time of writing this article, there are 91 accounts from before the attempt to deplatform anti-vaccine sentiment that are still on the Facebook (Meta) platform, with 56.04% of them being anti-vaccine pages (no. followers: avg. = 7,831, min. = 60, max. = 97,226) and 43.96% pro-vaccine pages (no. followers: avg. = 28,157, min. = 68, max. = 226,242).

After February 2019, during the pandemic, there was a shift in the number of vaccine pages: 20 anti-vaccination pages and six pro-vaccination pages were removed. However, the most staggering change was in the number of followers: there was a drastic decrease in the number of followers to anti-vaccination pages and increase in the number of followers of pro-vaccination pages. The sharp drop in anti-vaccine followers suggests that influential pages were deplatformed from Facebook (Meta), which is consistent with [22]. The increase in the average and maximum values of pro-vaccination pages suggests that more followers joined those discussions. Tables 3 and 4 show stigmatised sentiment as a share of the total data in response to both COVID vaccine posts and disproving COVID vaccine conspiracy posts.

**Table 3:** Datasets featuring COVID Vaccine Stigmatised Posts (anti-vaccine sentiment) and proportion of stigmatised sentiment in comments to those posts

Dataset description					Engagement value, posts			Engagement value, comments		
Reddit, twitter, youtube	# Posts	# Comments	Posts %	Comments %	Min	Max	Average	Min	Max	Average
Stigma	47	1975	100.00	21.31	300	194305	7726	− 62	1751	7.51
Not stigma	0	6406	0.00	72.35	0	0	0	− 37	2576	8.03
Undefined	0	473	0.00	5.34	0	0	0	− 26	631	5.42
Total	47	8854	0.53	99.47	300	194305	7726	− 62	2576	7.78

**Table 4:** Dataset featuring Disproving COVID Vaccine Conspiracy Posts (pro-vaccine sentiment) and proportion of stigmatised sentiment in comments to those posts

Dataset description					Engagement value, posts			Engagement value, comments		
YouTube	# Posts	# Comments	Posts %	Comments %	Min	Max	Average	Min	Max	Average
Stigma	23	6669	38.98	21.35	− 253	57828	4842	− 35	9395	16.49
Not stigma	35	23071	59.32	73.87	− 757	151570	9362	− 57	9715	22.13
Undefined	1	1490	1.69	4.77	3420	3420	3420	− 70	8618	22.48
Total	59	31230	0.19	99.81	− 757	151570	7499	− 70	9715	20.95

The slightly higher engagement with stigmatised anti-vaccine posts in Table 3 can suggest that in-group support still prevails. Varied engagement with posts that try to disprove conspiracy, shown in Table 4, may suggest a diversity of users that read the content, including out-of-group members. Engagement value is a supplementary feature included in the dataset with the goal of understanding the impact of stigmatised and non-stigmatised, anti-vaccination and disproving COVID vaccine conspiracy sentiments. It is also the most viable and direct method of studying the impact of sentiment on social media platforms. The feature combines the following scores: downvotes and upvotes (Reddit), likes (Twitter, YouTube), dislikes (YouTube), comments (Reddit, Twitter, YouTube), retweets (Twitter). Negative engagement primarily stems from Reddit and YouTube platforms through downvotes and dislikes, respectively.

### Comment examples

To understand the content of each of the classes, replies to posts are presented in Appendix A and Table 5. Six replies to each post were randomly chosen from the COVID anti-vaccine dataset to show instances of stigma, not stigma, and undefined sentiment. Similarly, randomly chosen examples from the disproving conspiracy posts and their comments are presented in Table 5 and Section 4.2. COVID anti-vaccine posts are those discussing conspiracy topics and expressing fear about vaccination side effects. Conspiracy sentiment includes discussions of the primary agenda behind the vaccine, speculation over identification devices in the vaccine, labelling of the pandemic as a fraud meant to eliminate small businesses, declarations that there is a 5% death rate from the vaccine without factual evidence, and vaccine development with the purpose of speculative market index.

**Table 5:** **Comment Examples** to Posts/Videos that try to **Disprove COVID Vaccine Conspiracy** (YouTube)

Post	Label	Comment
Is COVID vaccination female sterilization? a doctor explains	Disproving **Conspiracy**	1. One of the first things the drug companies did was to seek immunity from legal liability. That pretty much told me everything I needed to know about the safety of the vaccine. They clearly don’t even believe it’s safe. 2. And in a year when women can’t get pregnant I guess they ’ll be a retraction to this video. My point is, how the F do you know if this is true or not and why chance it??!Remember the drug they gave pregnant women for naseousness that made all their adult children have discolored teeth? My point is, WE DONT KNOW FOR SURE.
Pfizer says there are no safety concerns about its vaccine	Disproving **Side Effects**	1. It takes decades to determine safety. You have ZERO recourse if you’re injured. It’s hard to prove injury and it could take decades for problems to show up. 2. They have no responsibility if there are issues so who cares what they say? They are saying yeah it’s great you should get it so you can pay them! Would you go get surgery and sign a legal contract saying the doctor is not at fault for anything that goes wrong during surgery and also the doctor can do whatever he wants with no repercussions...? These people that makes vaccines can literally put anything they want in the vaccine and you can’t blame them for anything they have full immunity.
A Doctor discusses COVID vaccine safety	Disproving **Serious Side Effects**	1. Are they going to count every death within 28 days of the vaccine as they did with a covid diagnoses? If not, why not? 2. Hi. My question is if there would be large scale of epigenetic changes in the cell. Especially in the B cells, T cells? Immunoglobulin gene expression is vital, but my problem is that I dont know how much it would affect the human body. My theory is that the ammount of mRNA can change the gene expression in some cells. This way creating a difference in the cell population and leading to a cancerous process. My second point is that the mRNA would turn on some genes, enzymes too extensively. For example what if someone has a hidden auto immune disease: we turn on their genes, but some of their enzymes cant remove the histone mods. I also heared that the Pfizer vaccine doesnt change our genetic material. But Pfizer also has polyethylene glycol. I belive it could change the stability of guanine, quadruplexes. Probably this could cause cancer too.

The replies to COVID vaccine stigma posts that carry stigma sentiment (Appendix A) present the following main topics: i) agenda imposed by WHO, ii) Bill Gates, iii) the CDC, iv) population control, v) sterilisation, vi) becoming a lab rat, vii) denying the existence of a vaccine to prevent COVID, viii) calling the COVID pandemic imaginary, ix) demagoguery to reject the vaccine, x) death as a COVID vaccine side effect, xi) population control, xii) mark of the devil, xiii) agenda that has been forced by big corporations, xiv) people who get the vaccine will die, xv) calling the vaccine a murder weapon, xvi) announcing the existence of Microsoft microchips in the vaccines, and xvii) claims that the people who run the world are holding back the vaccines.

Not stigmatised replies to COVID vaccine stigma posts had the following topics: i) gratitude for the content posted, ii) explanations of what 95% vaccine effectiveness means, iii) expressions of worry about being unable to say no to the vaccine, iv) explanations about vaccine trials, v) suggestions of resources with factual evidence on the science behind the vaccines, vi) asking constructive questions, and vii) discussions of personal experiences. In addition, background was often given for the information provided.

Replies that were labelled as undefined (neither sentiment was identified) were i) asking rhetorical questions, ii) providing puzzling statements that can be interpreted as both carrying and not carrying stigma sentiment, and iii) comments hinting at a vaccine agenda, making a joke about it, or asking a question in order to understand the situation better. Discussions about serious side effects included suspicion that the vaccine was not properly tested or questioning vaccine trials, expressing fear of making it mandatory, and distrust in the effectiveness of the vaccine due to its speedy development.

Disproving COVID vaccine conspiracy posts were focused on disproving unconventional falsehoods, such as female sterilisation and challenges to concerns about the vaccines’ safety. Replies to those posts are displayed in Table 5. Replies that carry stigma sentiment exhibited the following main topics: i) drug companies being protected against legal liability, ii) uncertainty in relation to pregnant women and the long-term effect on their children, iii) beliefs that safety takes decades to determine, iv) “anything” can be placed in the vaccines, v) no responsibility for serious side effects, vi) lack of information about possible lethal side effects 28 days after the vaccination, and vii) the probability that mRNA vaccines lead to cancer and changes in genes.

### Classification models

Most comments correspond to the propagated label (stigma, not stigma, undefined), but some comments were misclassified. Before evaluating how well the models would perform, the text was split into bi-gram features in order to receive more meaningful segments of the data that would potentially lead to a more straightforward interpretation. Then, the score for each bi-gram unit was calculated to establish its importance in the corpus. Terms that appeared in fewer than five documents (posts/comments) were ignored. Traditional models were applied to the data, and the results were compared with pre-trained deep learning models.

Logistic regression can achieve comparable or better classification results on simpler tasks than can neural networks. However, the former can skew the result for the majority class on imbalanced data. Therefore, parameters need to be modified to take skewed distribution into account. Support vector classification is a superior technique to naive Bayes for text classification tasks. It achieved a better performance than logistic regression or naive Bayes; it also does not require tuning of the parameters. Moreover, random forest classifier (balanced subsample) is better suited for the classification task on an imbalanced dataset, because the undefined class is much smaller than the stigma and not stigma classes.

CNN is a good technique for some image recognition tasks; however, it can lead to over-fitting in text classification. To test the model’s performance and accuracy of the propagated labels, classification was performed on sub-samples of the dataset that had not been used for training of the algorithm. This resampling technique (bootstrapping) divided the dataset into B samples of identical size with replacement [53]. A separate model was built on each of the samples, yielding an n number of classifications, and bootstrapping eliminated the challenge of over-fitting. As a result, CNN with pre-trained word vectors achieved a very good classification result.

Deep Learning (Table 6) shows significant improvement in *F*1 values ($\bar {X} = 0.75$, *S* = 0.011), *tStat* = − 5.94, one-tail P 0.000 (T≤t) and two-tail P 0.001 values (T≤t) when comparing with traditional models (Mean ($\bar {X}$)= 0.67, Standard Deviation (*SD*) = 0.032).

**Table 6:** Test accuracy on classification task

Label spreading − COVID vaccine stigma	Accurracy	*F*1 score	Precision	Recall
TF-IDF, N-grams +Logistic regression	0.746	0.698	0.718	0.746
+Linear SVC	0.731	0.712	0.710	0.731
+MultinomialNB	0.732	0.649	0.680	0.732
+MLP (Multi Layer Perceptron)	0.707	0.662	0.661	0.703
+BalancedBaggingClassifier	0.612	0.639	0.695	0.617
+RandomForestClassifier (Balanced Subsample)	0.757	0.712	0.712	0.739
+RandomForestClassifier Balanced (class weight balanced, in favour of minority class)	0.622	0.649	0.715	0.622
+CNN (Glove.6B.50d) [57]	0.754	0.752	0.751	0.756
+CNN(Glove.42B.300d) [58]	0.762	0.757	0.752	0.766
+CNN(Glove.840B.300d) [59]	0.755	0.743	0.728	0.774
+CNN (FastText WikiNews− 300d − 1M) [60]	0.767	0.764	0.759	0.774
+CNN (FastText Crawl− 300d − 2M) [61]	0.763	0.756	0.744	0.781
+CNN (ELMoWiki20191024d) [62]	0.758	0.731	0.712	0.768
+CNN (GensimSkipGram300dWiki2019) [63]	0.750	0.746	0.739	0.759

All models were evaluated ten times by boostrapping on COVID vaccine stigma posts and their comments. The mean of achieved accuracy is reported for each model. CNN significantly outperformed baselines (traditional models), as per a paired sample t-test (*p* < 0.05), assuming unequal variances

Similarly, in the disproving conspiracy data (Table 7), deep learning outperformed traditional models. The *F*1 measure is much higher when comparing the performance of deep learning models ($\bar {X} = 0.79$, *SD* = 0.003), *tStat* = − 4.06, one-tail P(T≤t) 0.003, two-tail P(T≤t) 0.006) with the performance of traditional models ($\bar {X} = 0.73$, *SD* = 0.041). The null hypothesis should be rejected, as the classification accuracy of deep learning models is substantially higher than the accuracy of traditional models, which answered Q2.

**Table 7:** Test accuracy on classification task

Label spreading − disproving COVID vaccine stigma	Accurracy	*F*1 score	Precision	Recall
TF-IDF, N-grams + Logistic regression	0.785	0.751	0.769	0.785
+Linear SVC	0.782	0.768	0.766	0.782
+MultinomialNB	0.759	0.684	0.756	0.759
+MLP (multi layer perceptron)	0.736	0.745	0.746	0.739
+BalancedBaggingClassifier	0.655	0.678	0.734	0.651
+RandomForestClassifier (balanced subsample)	0.794	0.768	0.777	0.796
+RandomForestClassifier Balanced (class weight balanced, in favour of minority class)	0.655	0.686	0.758	0.661
+CNN(Glove.6B.50d) [57]	0.801	0.794	0.787	0.807
+CNN(Glove.42B.300d) [58]	0.796	0.789	0.780	0.807
+CNN(Glove.840B.300d) [59]	0.796	0.791	0.784	0.804
+CNN(FastText WikiNews− 300d − 1M) [60]	0.791	0.783	0.775	0.801
+CNN(FastText Crawl− 300d − 2M) [61]	0.792	0.788	0.781	0.803
+CNN(ELMoWiki20191024d) [62]	0.796	0.789	0.782	0.803
+CNN(GensimSkipGram300dWiki2019) [63]	0.796	0.790	0.783	0.804

All models were evaluated ten times by boostrapping on Disproving COVID vaccine stigma posts and their comments. The mean of achieved accuracy is reported for each model. CNN significantly outperformed baselines (traditional models), as per a paired sample t-test (*p* < 0.05), assuming unequal variances

An *F*1 score of 0.764 (as seen in Covid Vaccine Stigma, Table 6) was achieved with a CNN that was pre-trained on FastText WikiNews-300d-1M. FastText WikiNews-300d-1M contains 1 million pre-trained word vectors with 300 dimensions (features) that was trained on the Wikipedia 2017 data, UMBC webBase corpus, and statmt.org news dataset.

An *F*1 score of 0.794 (as seen in Disproving COVID Vaccine Stigma, Table 7) was achieved with a CNN that was pre-trained on Glove.6B.50d. Glove.6b.50d contains 400,000 pre-trained word vectors on Wikipedia 2014 data and Gigaword5 files. It also contains 6 billion tokens, 400,000 of uncased vocabulary, and 50 (features) dimension vectors. Evidence that the CNN model achieved *F*1 precision of 0.794 on the identification/classification task suggests that the propagation task (on the stigma, not stigma, and undefined labels) and model for identifying subtle stigma sentiment were implemented effectively and perform well.

### Features

#### LIWC variables

Prior to the development of LIWC, Walter Weintraub hand-counted people’s words in medical and political speeches and linked them to emotional states of the person [64]. Weintraub was fascinated by how people use language. He associated an impulsive personality trait and binge eating disorder with frequent used words “but”, “nevertheless”, “however”. People with those disorders act impulsively, and it is reflected in their speech when they use such terms to try to remedy the consequences of an impulsive action by taking back the statement. Similarly, persons with compulsive repetitive behavior try to justify such acts using expressions such as “because”, “therefore”, and “in order to” [64].

Weintraub’s method of analysis looked for verbal categories such as qualifiers (“think”, “kind of”, and other filler words) that are inversely related to preparation; retractors suggest difficulty in adhering to previous decisions (“however”, “but”); personal pronouns present an individual (“I”), a mutual course (“we”), and a more passive speaker (“me”); negatives suggest stubbornness, opposition, or the use of coping mechanisms (“not”, “never”, and “nothing”); and adverbial intensifiers produce dramatic effect and are used by teenagers more than other age groups (“very”, “really”, “so”, “such”) [64].

Furthermore, verbal categories were also associated with personality traits. Decisiveness was connected with high frequency use of qualifiers, an angry disposition was associated with an increase in negatives, as much as five times that of normal speech, an increase in the use of rhetorical questions and direct references [64].

LIWC (“Luke”) was developed similarly, with the initial goal of efficiently counting words in psychologically or grammatically-relevant categories across multiple text files. Central to the analysis are LIWC dictionaries with collections of words that define categories [65]. All the relevant categories are listed, and the percentages for each category are given per post/comment, based on the total number of words in post/comment (analysis concerned social media data). Some LIWC categories are rather straightforward, such as articles, which consists of three words (“a”, “an”, “the”), whereas other social and emotional processes are more complex, such as where three researchers had to agree on the assignment of words to those categories [65].

From its first version, LIWC 1997 [66], to the LIWC 2015 [67] version, LIWC software studies social, psychological, and linguistic processes in an efficient way. The LIWC feature analyses based on a written text can reveal a lot about an author or historical figure quickly and correctly, adding to a description by historians. Furthermore, the latter can also carry bias.

For example, the use of more tentative language, such as filler words, suggests that a person is uncertain/insecure about the topic. Negative emotions, death references, and first-person singular can suggest that a person is depressed, with suicidal thoughts [65].

There are various research articles that successfully apply LIWC features to perform correlation, classification type tasks [28, 68, 69], and prediction type tasks [70].

Schizophrenia stigma in [28] was studied with 27 LIWC features and was associated with social processes, humans, death, and anger. Similarly, character traits such as narcissism have been analysed with 72 linguistic features from LIWC 2001, using weighted Pearson’s correlation technique [68]. The results showed a positive connection between narcissism, sexual references, swear word use, and a negative association with anxiety. LIWC features also helped to classify positive and negative sentiment from social media opinion posts [69]. High classification accuracy scores on the task were achieved with the following features: psychological processes, relativity, and personal concern.

Furthermore, prediction of the final course performance based on written self-introductions by students was described in [70]. Here, 84 of the LIWC features were gradually reduced to 20 based on the correlation with the final grade. Analysis was based on 321 written self-introductions and concluded that egocentrism and acting-in-the-present were linked with poor performance on the exam (prevalence of personal pronouns, use of verbs, and present tense words).

The current study includes features from the LIWC 2015 version, together with five other features that were defined in the research and are presented in Appendix B. The variables in Appendix B help us to understand the social, emotional, and linguistic composition of the COVID vaccine stigma sentiment with the most relevant features of the model discussed in Section 4.4.2.

#### Features of the model

The 30 most significant features in Tables 8 and 9 were derived from variables in Appendix B and are based on ANOVA F-test and RFE ranking. The latter identified the informative features, and the ANOVA F-test determined whether there was any statistically significant difference between mean values of features and annotation labels (classes) and how well a given feature discriminated between multiple classes.

**Table 8:** Anti-COVID-Vaccine Sentiment Posts and Comments (Reddit, Twitter, YouTube)

Features	Ratio of variance (features and labels)		*Z*-score: standard error of the MEAN		
	Anova F-score	RFE ranking	Stigma	Undefined	Not stigma
Engagement	0.67	1	− 0.2336	− 1.1706	− 0.2845
No. characters	300.29	1	20.1163	− 9.0579	− 8.7083
Sentiment score (polarity)	35.76	1	− 7.3995	0.2776	4.0332
Subjectivity score	17.85	1	3.3616	− 4.8973	− 0.5358
Word count	342.46	1	21.4970	− 9.2042	− 9.4352
Analytical thinking	9.1	1	− 3.1982	− 1.7073	2.2397
Authentic	4.44	1	− 2.1890	− 1.2813	1.5636
Words per sentence	74.44	1	7.1972	− 9.6303	− 1.3794
Words > 6 letters	27.22	1	− 5.1200	− 3.6369	3.8311
% words captured by the dictionary	48.33	1	7.5171	3.5624	− 5.1419
Function words	72.04	1	10.1683	− 4.2787	− 4.4833
Article	49	1	7.9747	− 4.8696	− 3.1048
Verbs	13.19	1	3.6644	2.3829	− 2.6822
Focuspresent (today, now)	21.81	1	5.5583	1.0652	− 3.3757
Emotional tone	24.5	35	− 5.9594	− 0.8417	3.5377
They	18.08	42	4.7497	− 3.2185	− 1.7627
1st person singular: I	6.08	8	3.0718	− 0.5034	− 1.5688
Auxverb (may, must)	14.83	2	4.7836	− 0.3496	− 2.5611
Conjunctions (but, whereas)	20.71	12	5.1905	− 3.2018	− 2.0120
Positive emotions (happy, good)	23.91	18	− 5.2869	4.0301	1.8405
Negative emotions (hate, enemy)	22.88	26	3.2534	4.9939	− 3.1634
Anxiety (afraid, tense)	9.24	63	3.7865	− 0.5490	− 1.9533
Anger (hate, kill)	8.89	57	2.6554	2.4689	− 2.1453
Perceptual processes (touch, listen)	14.54	24	− 4.7222	1.1790	2.3016
Biological processes (eat, blood)	7.05	19	3.2978	− 0.7418	− 1.6295
Health (clinic, flu)	13.5	38	4.3073	1.0769	− 2.6843
Risk (danger, doubt)	14.26	52	2.6427	3.8841	− 2.5228
Work (work, boss)	8.81	31	− 2.7687	− 2.2940	2.1607
Swear words (damn, shit)	6.66	62	2.6038	1.7009	− 1.9079
Netspeak (lol, thx)	7.02	46	− 3.2335	1.1757	1.4759

**Table 9:** Disproving COVID-Vaccine Conspiracy Posts and Comments (YouTube)

Features	Ratio of variance (feature and label)		*Z*-score: standard error of the MEAN		
	Anova F-score	RFE ranking	Stigma	Undefined	Not stigma
Engagement	2.25	1	− 1.8802	0.3061	0.9331
No. characters	462.2	1	26.1821	− 8.3246	− 11.9612
Sentiment score (polarity)	53.75	1	− 9.1252	0.0830	4.8850
Word count	485.76	1	26.8784	− 8.1634	− 12.3765
Analytical thinking	164.08	1	− 8.9897	− 13.2872	8.2100
Words per sentence	200.87	1	15.4858	− 11.2705	− 5.4617
Words > 6 letters	143.15	1	− 13.3460	− 5.6198	8.6036
% words captured by the dictionary	285.26	1	16.9273	11.3958	− 11.9969
Function words	240.8	1	16.6547	8.5509	− 11.1274
Pronoun	153.11	1	8.4750	13.0217	− 7.8658
Verbs	233.63	1	9.9696	16.4338	− 9.5365
Social processes (talk, friend)	92.68	1	3.1265	12.3041	− 4.8078
Cognitive processes (cause, ought)	23.65	1	5.2994	2.6110	− 3.5128
Focuspresent (today, now)	205.87	1	11.2223	13.7693	− 9.5329
Emotional tone	106.84	32	− 12.8242	− 0.0654	6.9115
Ppron (them, itself)	84.19	6	4.8735	10.7301	− 5.3471
They	61.4	37	9.8048	− 1.5425	− 4.8795
1st person singular: I	1.79	9	6.6539	6.5443	− 5.2406
Auxverb (may, must)	171.85	4	12.5059	9.9084	− 9.2418
Negate (not, never)	37.45	16	5.1319	5.5652	− 4.1734
Positive emotions (happy, good)	136.87	12	− 13.0077	8.9372	4.7223
Negative emotions (resent, enemy)	169.86	15	15.8379	1.9796	− 9.0183
Anxiety (afraid, tense)	15.35	67	4.8959	− 0.2061	− 2.5799
Anger (rage, hurt)	59.82	53	8.8268	3.2141	− 5.5625
Discrepancy (should, could)	50.61	33	7.9232	3.4318	− 5.1320
Differentiation (hasn’t, else)	40.96	19	7.9521	0.0898	− 4.2982
Perceptual processes (touch, listen)	70.22	17	− 10.4499	0.3849	5.5206
Risk (danger, doubt)	29.44	52	6.7911	− 1.2274	− 3.3393
Focusfuture (will, soon)	51.16	43	8.6456	1.4464	− 5.0159
Swear words (damn, shit)	66.96	63	10.1746	− 0.0334	− 5.4618

The *z*-score indicates how much the labelled classes can vary from the population mean. Certain features show polarised development of stigmatised comments versus not stigmatised comments for both the COVID vaccine stigma and disproving COVID vaccine conspiracy datasets.

Sentiment score (polarity on negative and positive sentiment), subjectivity, and engagement are additional features that are not part of LIWC variables.

Sentiment feature shows negative score for stigmatised comments and positive for not stigmatised. Subjectivity is naturally higher for stigmatised content, and is also confirmed by the findings in Tables 8 and 9.

Stigmatised sentiment is expressed in lengthier sentences/comments, which is presented through the high positive *z*-scores of words-per-sentence feature. Stigmatised sentiment is also seen in lengthier posts/comments (word count/no. characters feature). There is more stigmatised communication than not stigmatised. Function words that reflect the attitude or mood of a speaker are more frequent in stigmatised comments, which focus on the present time and exhibit the characteristics of negative emotions (such as anxiety and anger) and the use of swear words. Prevalence of present tense suggests greater psychological connection and continuation of the concern.

References to risk and danger are common, as are references to out-groups (“they/them” vs. “us”). Stigmatised sentiment is expressed with less emotion, which can suggest lesser involvement with the topic and features excessive use of auxiliary verbs (“may”, “must”, “should”).

Conversely, perceptual processes (selecting, organising, and interpreting information) and work references are common in not stigma sentiment.

Not stigmatised comments/sentences are succinct, but they employ lengthier informal words, which suggests that more complex words are used. Moreover, not stigmatised sentiment is expressed in an emotional, authentic, and positive tone that is simultaneously analytical. Emotional tone can suggest greater immersion in the topic. In contrast to stigmatised sentiment, risk, danger, anger, and references to health, anxiety, and other negative emotions, such as swear words, are rare in not stigmatised sentiment.

The engagement feature was log normalised to remove skewness from the highly variable data and is based on downvotes, upvotes, likes, dislikes, comments, and retweets. Engagement is important for the study as it can show different levels of participation in vaccine discussions. The RFE ranking deems the feature to be relevant for stigma detection. However, *z*-score and ANOVA F-score did not detect any significant variances in engagement across stigma class labels.

**Fig. 3:**
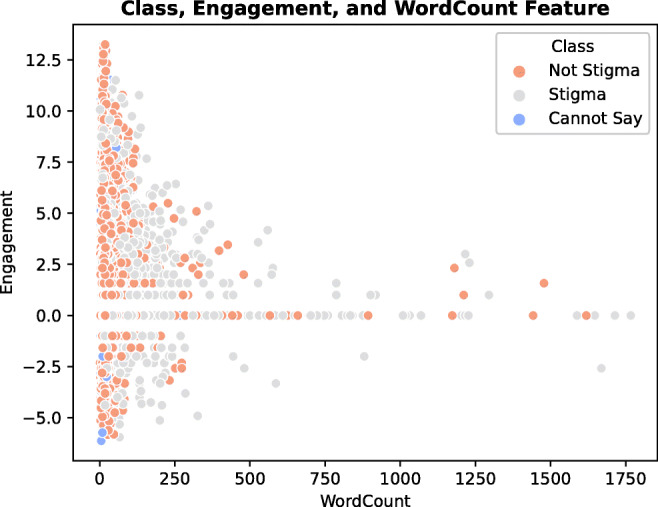
Stigma and engagement

Unsupervised learning K-means clustering can serve as an additional visual interpretation of the features of the model. According to the distribution of the data in Fig. 3, stigmatised posts have higher word counts/are lengthier than not stigmatised posts, which is supported by the *z*-score findings in Tables 8 and 9. Stigmatised posts/comments receive mixed response (engagement), similar to not stigmatised posts; however, some show especially high engagement. From the observation of the study, the connection between engagement and stigma depends on the context. For example, in the in-group anti-vaccine discussions, stigmatised posts received more attention and consequently reported high positive engagement. Conversely, not-stigmatised posts are more emotional and authentic, using informal language, which can draw attention to the post in other contexts. However, further discussion on the topic of engagement is outside the scope of the current research and will be discussed in future work.

### Visual analyses: co-occurance network

To further visualise the comment responses to COVID vaccine stigma and disproving conspiracy posts, a co-occurrence network of words was applied with term frequency (69) and document frequency (1). To measure the strength of edges, the Jaccard coefficient was applied with the top 77–105 words presented. Darker lines and higher coefficients show stronger edges (coef. ≥ 0.1).

**Fig. 4:**
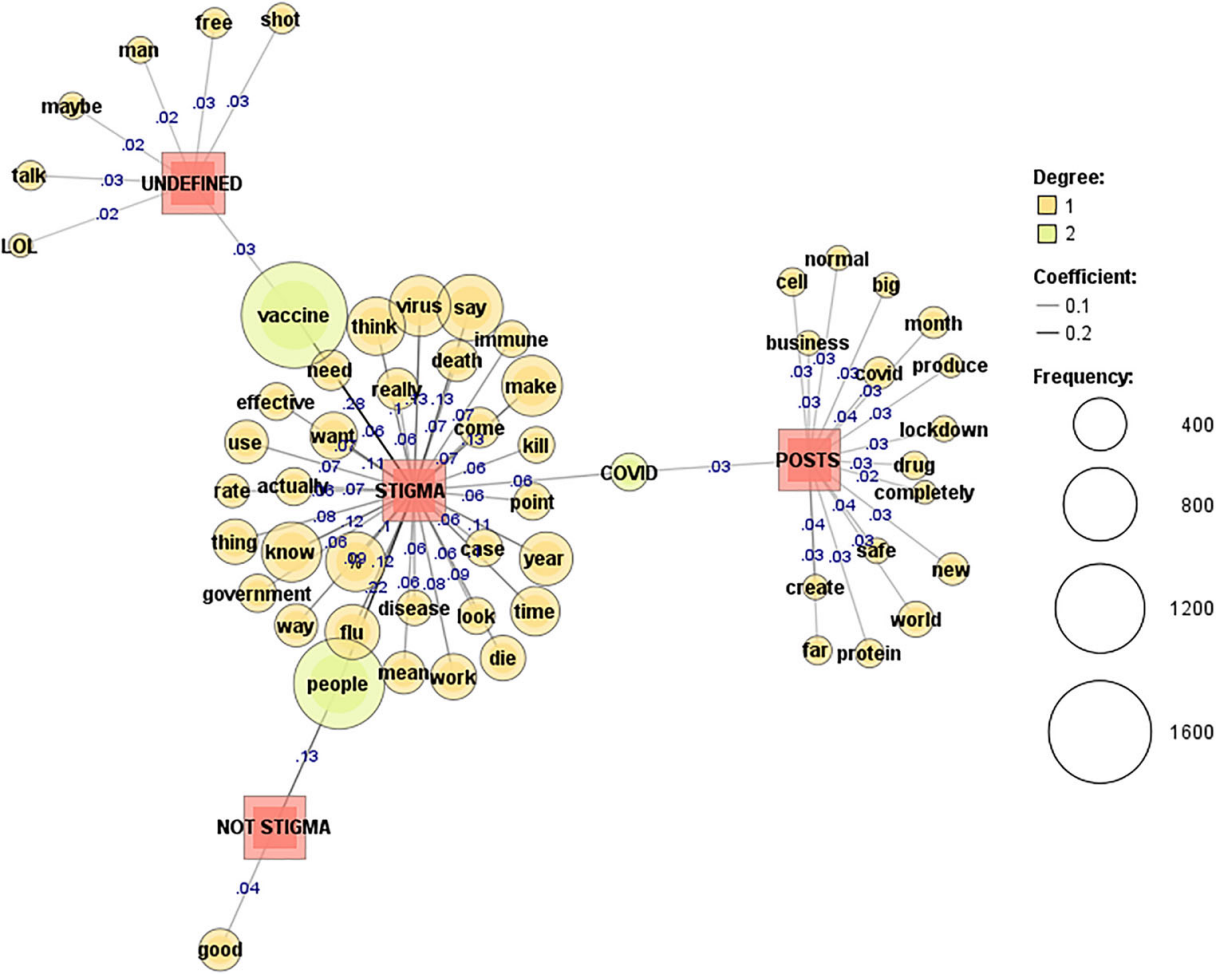
COVID vaccine stigma: Reddit [71]

The stigmatised Reddit posts in Fig. 4 show representative words such as “big”, “business”, “covid”, and “produce”, suggesting a fair share of the discussion is attributed to big business and its role in the pandemic.

“COVID” is characteristic of both stigmatised posts and comments. Stigmatised comments echo some of the sentiment from the posts with references to “government”, “kill”, “covid”, and “vaccine”. Central in the discussions is criticism of governments and warning against side effects of the vaccines. References to “kill”, “die”, and “death” under the topic of vaccines suggests fear and depressive moods of the people who wrote the comments.

**Fig. 5:**
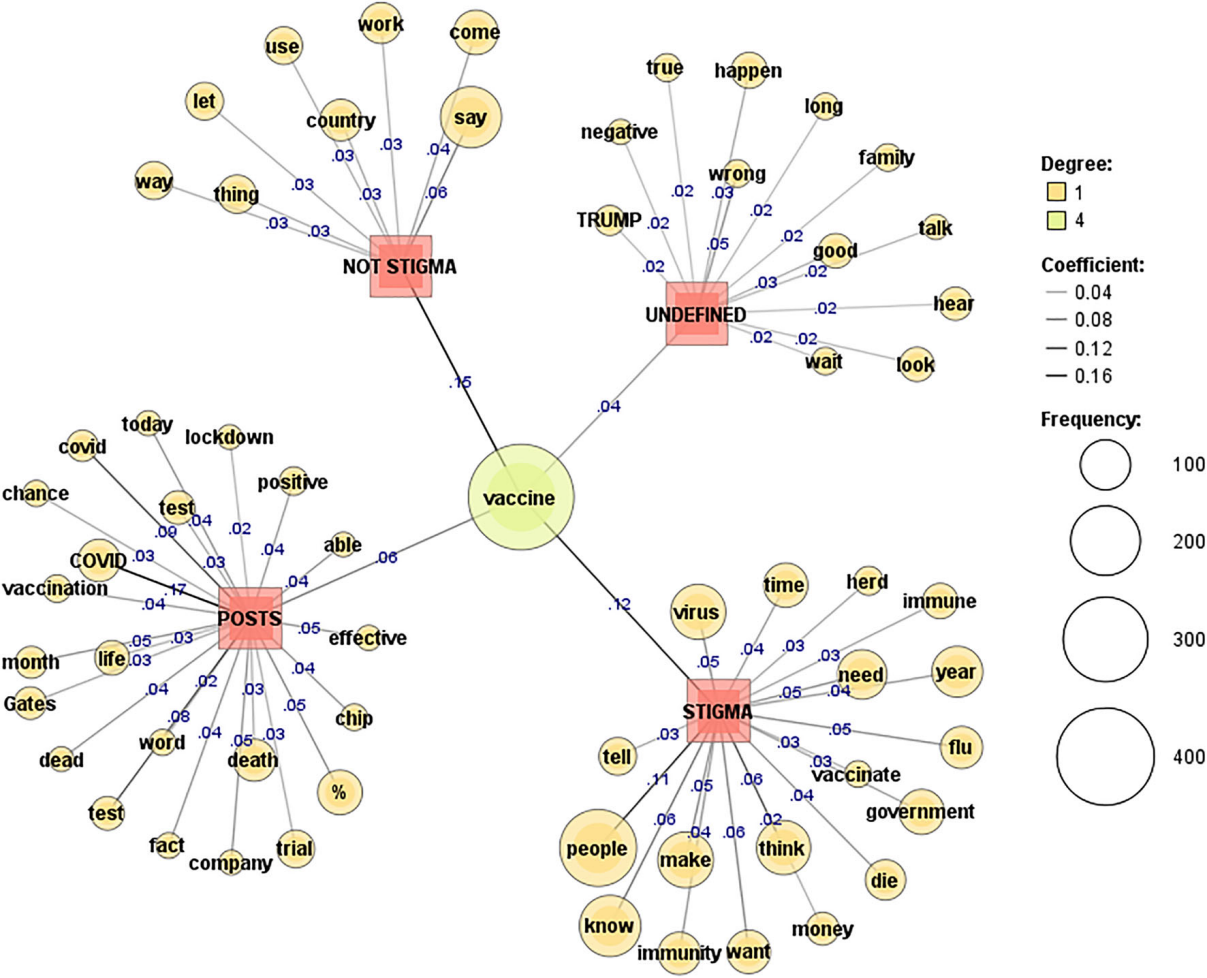
COVID vaccine stigma: Twitter [71]

The mentioning of “vaccine” is particularly frequent in stigmatised and not stigmatised comments on Twitter (Fig. 5). Twitter posts discuss COVID vaccine “effectiveness”, “Gates”, “chip”, and include several references to “death”. Stigmatised Twitter comments refer to “government”, “people”, “know”, “virus”, and “money”.

**Fig. 6:**
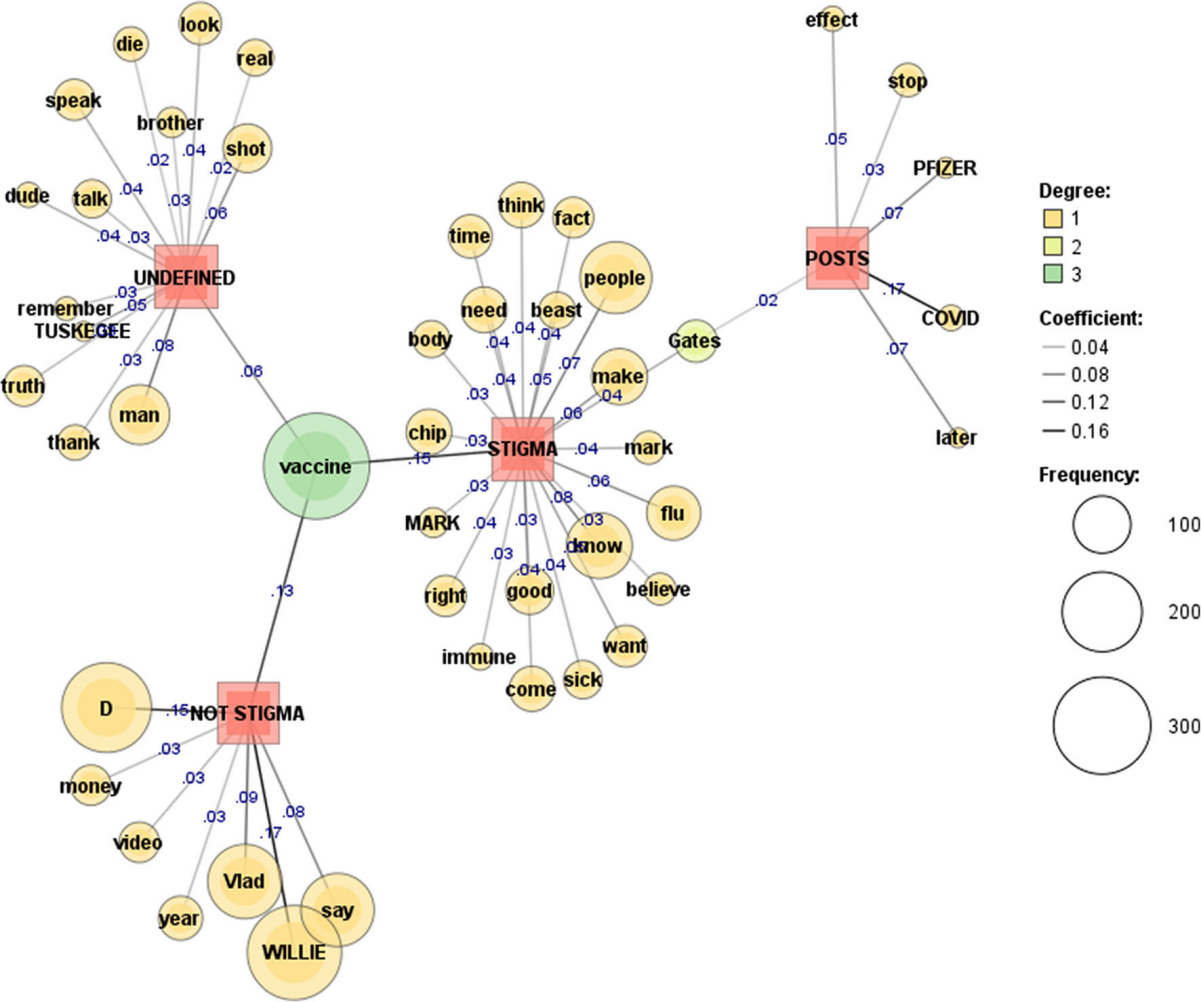
COVID vaccine stigma: YouTube [71]

The YouTube anti-covid vaccine posts shown in Fig. 6 make references to “Pfizer”, “Covid”, and “Gates”. All comments mention vaccine to a lesser or greater degree; stigmatised comments also make references to “Gates”, “mark”, “beast”, and “chip”.

**Fig. 7:**
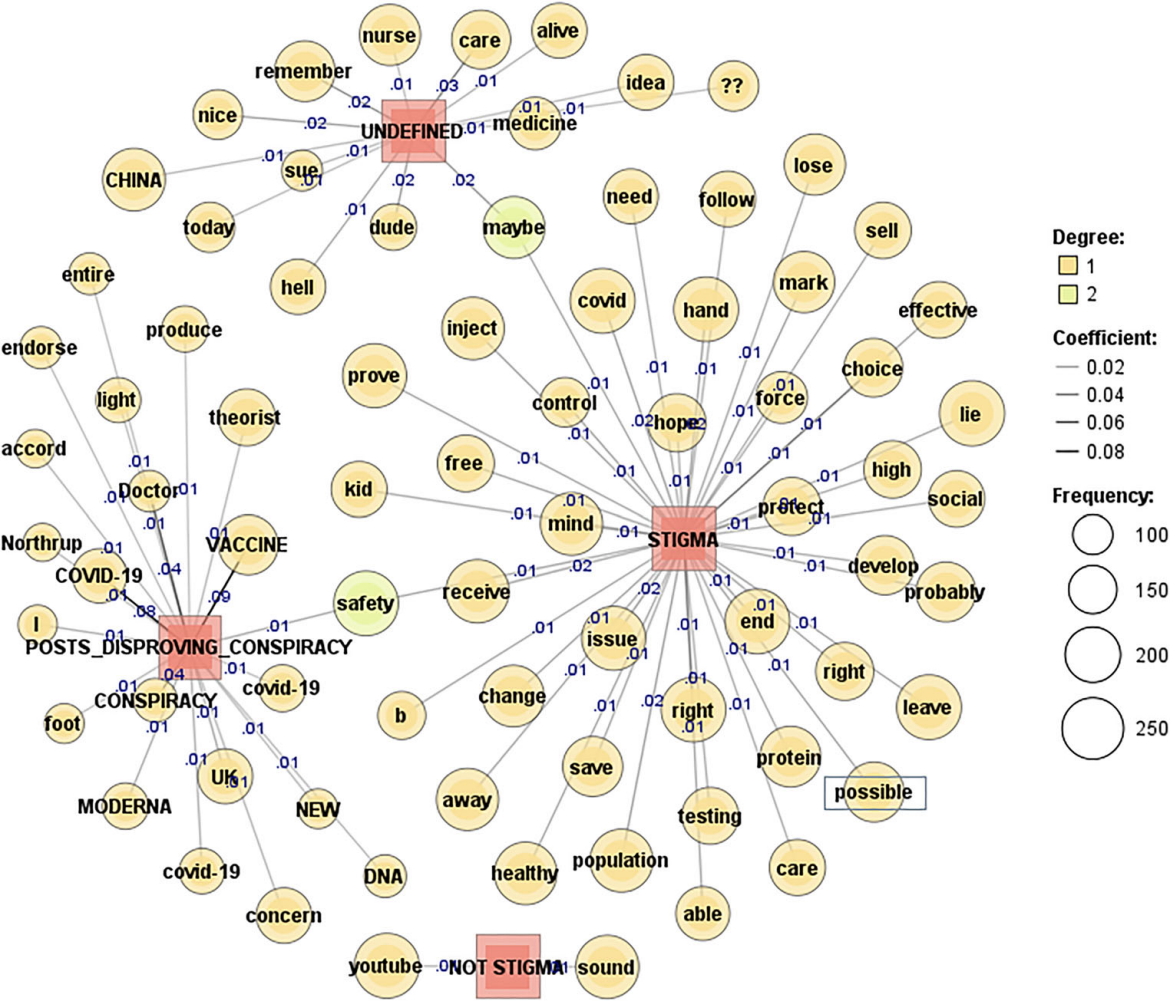
Disproving COVID vaccine stigma: YouTube [71]

The co-occurrence network of words, at times, provides us with an ambiguous, breviloquent idea about the main sentiment and topics discussed within a certain context. Correspondingly, sentiment gleaned from visual analyses provides us with a vague yet apropos conceptualisation of the stigma, not stigma, and undefined classes. The posts shown in Fig. 7 discuss vaccine conspiracy and alleged effects of the new vaccines, such as DNA-related risks, along with other concerns about side effects connected with the Moderna vaccine. Doctor Northrup—a known figure in the anti-vaccine movement—is frequently mentioned in the posts trying to disprove a COVID vaccine conspiracy. Stigmatised responses mention population control, forced practices, and appeal to freedom of choice in the arguments. Stigmatised comments also question the effectiveness of the vaccines and suggest that the vaccines did not go through proper development and testing procedures in such a short time frame.

## Discussion and conclusion

This paper presented a computational model for identifying COVID vaccine stigma across social media platforms and addressed how to build such a model. To the best of the author’s knowledge, this is the first time a computational model of vaccination discourse has been designed and the first research on COVID vaccines based on four social media platforms. Numerous annotators were involved in the process and several approaches were tested before each comment was annotated; consequently, labels propagated to a larger dataset. The goal of the model was to test how robust and reliable the model would be once classes were propagated from the vaccine discussions on Facebook (Meta) dataset to the COVID vaccine discussion on Twitter, YouTube, and Reddit dataset.

Without a rigorous impartial annotation process, annotation scheme, the identification of such a nuanced concept as stigma would be unlikely, and the identification of sentiment would be completed with much less accuracy. All classification models achieved high levels of accuracy, but there is a statistically significant computational advantage in the performance of deep learning models. The deep learning models with pre-training significantly outperformed traditional classification models and successfully identified stigmatised sentiment.

Features of the stigma and not stigma classes are quite indicative of the annotation label assigned. In particular, stigma sentiment in COVID vaccine discussion is expressed in the following characteristics traits: i) lengthier sentences, ii) showing negative sentiment, emotions of anxiety, anger, and those connected with risk, as well as the use of swear words, iii) is less analytical, iv) uses more auxiliary verbs such as “must”, “should”, and “can”, and v) employs a relatively reserved tone. Prejudiced sentiment leads to ignorance, hostility, and barriers to communication: “Erroneous ideas, Spinoza observed, lead to passion—for they are so confused that no one can use them as a basis for realistic adjustment. Correct and adequate ideas, by contrast, pave the way for a true assessment of life’s problems” [72].

Therefore, neutral and not stigmatised sentiment is preferable, especially with polarised topics such as vaccines. This calls for the characteristics of i) shorter sentences, ii) more analytical features, iii) an authentic tone, iv) positive emotions void of anxiety, anger, and risk, with no use of swear words, and v) an informal tone, void of discrepancy, and differentiation. Stigmatised sentiment in COVID vaccine discourse does not lead to negative engagement with the content and the study did not find engagement to be a relevant feature in identifying stigma sentiment in COVID vaccine discourse. This could be explained by the mixed reaction to public posts/comments from anti-vaccine and pro-vaccine communities. Stigmatised anti-vaccine posts/comments might be considered engaging among like-minded in-group members, but might receive negative reactions from the pro-vaccine community and show neutral engagement on the balance.

This study found that anti-vaccine sentiment is often present in the comments as responses to disproving conspiracy posts. This finding is unexpected, given that previous work discovered antagonists (anti and pro-vaccine movement) concentrated primarily within their own public groups on Facebook (Meta), with homogeneous position on the topic of vaccines and abstinence from out-of-group activity [14, 24]. Such contradictory evidence may be in connection with the special circumstances of the COVID pandemic, where COVID anti-vaccination pages and posts were removed whereas some groups banned across social media platforms. In response, the COVID anti-vaccine movement rebounded by moving to pro-vaccine channels, argued conspiracy theories and general stigma beliefs in response to statements attempting to disprove them. Some form of contact between COVID pro-vaccine and COVID anti-vaccine groups had thus been established.

Government attempts to de-platform the anti-vaccine movement did not succeed, but, instead, led to involuntary contact of the two groups. However, whether it was the right type of contact to reduce prejudice and prevent vaccination conspiracy theories, at least on a smaller scale, or if it provoked an even greater divide should be examined further. According to Gordon W. Allport, prejudice results from the lack of dialogue, lack of contact [5], and the antipodal stance can arguably be lessened when polarised groups are brought together [9, 72–77]. In his 1954 work, Gordon W. Allport also stated that prejudice between an in-group and an out-group may be reduced under certain conditions [72]. The effects of the contact will be enhanced if it is encouraged by law, customs, or given general conditions for the contact hypothesis to succeed: equal background, mutual goals, intergroup cooperation, and acknowledgement of authority that supports the interaction [72].

Elliot Aronson cultivated additional conditions: mutual interdependence, opportunity for frequent contact, and social norms that support such interactions [73]. Pettigrew et al. (2011) highlighted other positive outcomes of intergroup contact, such as greater trust and forgiveness of past transgressions [77]. Other researchers have indicated that effects generalise beyond immediate out-group members; are present across age ranges, genders and nations; and are related to not only ethnicity but also take place regarding healthcare and social issues [77]. Therefore, one can presume that the hypothesis generalises well for pro-vaccine and anti-vaccine groups. However, McClendon (1974) argued that one type of contact alone is not sufficient for optimal prejudice reduction and suggested a combination of Allport-Pettigrew theory and the theory of superordinate goal achievement [72, 77–79].

Unfortunately, all those special conditions seem to be very difficult without serious supportive initiatives. Moreover, there is also a number of authors who have argued that reduction in prejudice is possible only on a smaller scale [80, 81]. Amir (1969) argues the opposite effect from contact under unfavourable conditions [80]. Consequently, it can be a matter of future work to establish the optimal conditions for prejudice reduction and ways to create a constructive dialogue between anti-COVID and pro-COVID vaccine communities. Nevertheless, constructive dialogue is important due to opposing views in the emotionally charged case of anti-vaccine campaigns that continue to pose a challenge to the efforts of public health authorities.

The issue is not likely to subside by removing anti-vaccine groups from social media platforms, as those messages nevertheless find their way back, according to the findings in the current research and analyses from [22, 25]. Rifts between members of anti-vaccine, pro-vaccine movements, and polarised groups in the broader context, lead to irresolution, mockery, distrust, friction, antagonism, and destabilising situations in society as the long-term result. The findings in this research can guide the choice of impartial, unbiased communication features in the future where it can possibly motivate concordant action, successful execution of commitment to reduce the dissonance, and establish constructive dialogue between polarised vaccine groups.

## Data Availability

The data supporting the reported results can be obtained from the author upon reasonable request.

## Appendix A: Comment examples

**Table 10:** **Comment Examples** to **Covid Anti-Vaccine Posts** (Reddit, Twitter, YouTube)

	Posts		Comments		
Source	Stigmatised	Annotation	Stigmatised	Not stigmatised	Undefined
YouTube (Both)	The agenda behind Bill Gates vaccine and ID2020 : coronavirus conspiracies	**1. Expression that sustain hostility**: 1) Blame 2) Suspicion 4) Exaggeration **2. Expressions that sustain inconsistency and overgeneralisation**: 2) One-sided interpretation 3) Prediction 4) Guessing 5) Unsupported judgement or personal opinion / projection	1. End Fauci, End Gates, End the Fed, End the WHO, End the CDC, End the Tyranny!! 2. 1:15 Bill Gates ‘predictions’, 3:02 ID2020, 8:09 Bill Gates agenda, 8:56 population control, 14:11 dialectic.	1. Well done, an excellent video, THANK you! 2. It’s great to see you’re broadening your skills and presentations to yet more issues of concern for the world. We’re fortunate to have such a strong woman who does excellent research fighting for the People.	1. Let’s remember it: BGs father was head of planned parenhood. 2. So what is the connection between Trump and Bill Gates? Are they working together??
YouTube (Both)	Brazils Bolsonaro mocks possible side effects of Pfizer COVID-19 vaccine	**1. Expression that sustain hostility**: 3) Conflict (hate, fear), 4) Exaggeration, 5) Strong emotion, 9) Condescension. **2. Expressions that sustain inconsistency and overgeneralization**: 1) Inflexible unfounded	1. It’s dangerous we don’t know what’s in it. 2. Sterilization.	1. Vaccine should be mandatory for serious patients in the time of necessity not as precautionary needles. 2. Some common misconceptions: the 95% are the chance you have that the vaccine works. If it dis not work you	1. I love may president! 2. That man is promoting a genocide.
		over-generalization, 2) One-sided interpretation, 5) Unsupported judgement or personal opinion / projection, 7) Tabloid thinking, 8) Demagoguery.		still have your normal immune system. So the vaccine only makes it more secure.	
YouTube (Both)	COVID VACCINE skipping safety testing	**1. Expression that sustain hostility**: 4) Exaggeration, 5) Strong emotion, 9) Condescension **2. Expressions that sustain inconsistency and overgeneralisation**: 1) Inflexible unfounded over-generalization, 2) One-sided interpretation, 5) Unsupported judgement or personal opinion / projection, 7) Tabloid thinking”	1. “Sweden had the same information about the virus as the United States. Why then did Sweden not decide to commit suicide, but we did?” 2. The simple question is if you’re vaccine is so safe, then why are they trying to get a indemnification against being sued??	1. I’m not worried about not being allowed to yes to the vaccine I’m worried about not being able to say nonto a vaccine. 2. I’m in the trial! We’ll see next year if I’ve made any antibodies.	1. VAXXED. 2. FYI your thumbnail is very distorted.
Twitter (Hostility)	WHO are set to begin Covid 19 vaccine trials in our country. EVIL! Not only have our people been turned into Guinea pigs to test Gates’s killer vaccine but our leaders are also passing a law which will make the use of that evil vaccine compulsory.What a mess! I weep for Nigeria.	**Expressions that sustain hostility**: 1) Blame, 3) Conflict (hate, fear), 5) Strong emotion”	1. All Biafrans in that zoo nation must refuse these vaccines. When they can’t force you, they will try to entice you with money or food. Do not be fooled into becoming a lab rat for these people. You will spread the virus and you will die. There is no vaccine for Covid-19!!! 2. There are more of us than all of them including their military, police, and paramilitary put together. All we need to do is to put the word out there and instruct our people to completely reject the vaccines period.	1. More than 100 countries have joined the solidarity trial please read the Article. 2. I hope Buhari gets vaccinated first.	1. Even if I educate you about vaccines, you’ve already made up your mind. Remember you’ve been taking vaccines before now and your children will also take vaccines against common vaccine preventable childhood diseases. Have they had issues? 2. Then you should have no worries volunteering yourself and your family members take the trial vaccines as you know so much about vaccines abi?
Twitter (Overgeneralisation)	The flu vaccine has been about for about 80 year. It is only 50% effective. Flu kills 15000 people a year in England alone on average. They’ve had a long time to develop this vaccine and it is still pretty poor. Fancy that COVID one they have spent 6 months on?	**Expressions that sustain inconsistency and overgeneralisation**: 2) One-sided interpretation, 6) Dichotomization, 7) Tabloid thinking	1. Some ppl will die within days of receiving it. Better to die fighting their criminal underworld! 2. What if all this attention on the vaccine is a distraction? What would we see if we looked where they didn’t want us to look? Is the vaccine really a placebo and it will be our fear that kills us? The imaginary vaccine for the imaginary virus? A reason for the control passports.	1. I was going to say do some research, but you need some learning and understanding of viruses. Start with genetic shift and drift for flu. Not interested in a debate as I’m not an expert. 2. Ironically, I just finished booking my flu vaccine!	1. I’m sorry that happened. 2. Hell NO.
Twitter (Both)	Radio frequency (RFID) and near field identification chips could be put in the vaccine for COVID-19. What do you think about this?	**Expressions that sustain hostility**: 2) Suspicion, 3) Conflict (hate, fear) **Expressions that sustain inconsistency and overgeneralisation**: 3) Predicting, 4) Guessing, 5) Unsupported judgement or personal opinion / projection, 7) Tabloid thinking, 8) Demagoguery”	1. Nope...666 mark of the devil? Not for me! 2. Mark of the Beast technology Omar, gives them dangerous access to our lives.	1. That’s not a thing. 2. Why? What medical reason? Sounds like invasive action. Who gets to monitor the feed?	1. I am not getting any vaccine. 2. I already have an RFID/NFC chip, I dont want one from the government, I already have my own.
Reddit (Hostility)	Coronavirus is a scam to kill off all the small businesses so that only big corporations remain. In the U.S. there are around 40,000,000 small businesses. That means 12% of all people in the U.S. own some type of small business. We are now coming up on one year of this covid hoax clown world drama. You have seen literal sheep people BEGGING for a vaccine so that we can ’return to normal’. What they don’t realize is, it will stay the same even if there is a vaccine made from culture human fetus cells in a cow’s rectum. If this statement about the destruction of small businesses is is false, then the U.S. and news would be addressing this more as a huge problem. Instead, it’s barely even talked about! Even in the large big-box places, they are trying to condition people to not leave their couches and just order from online, treating customers like lepers, freaking out at customers, and having customers park their car in new world order online purchase spots, and have an employee shuttle the items	**Expressions that sustain hostility**: 1) Blame, 3) Conflict (hate, fear) 4) Exaggeration 5) Strong emotion”	1. It’s not a scam in the sense that the virus isn’t real, but yes you are 100% correct that it is being misused to push this agenda. 2. This is correct. Because once it’s all only large corporations, they know it all only be companies that will accept the mark. Also, they want as many people on UBI as possible so that you’re dependent on them for money and they can make you do whatever they want or else they’ll threaten to cut off that money.	1. I had to put you back to a positive upvote count. Not sure why you were downvoted for encouraging businesses to plan ahead for things like this. 2. Have you looked at excess deaths this year?	1. Meanwhile we’re still in lockdown. 2. SHHHHHHH let them be stupid.
	out. It’s crimes against humanity and it’s disgusting. And the policymakers are all bootlicking worms and cowards, and should be ashamed of themselves.				
Reddit (overgeneralisation)	A COVID vaccine with a 95% success rate definitely means that 5% of the people that get the vaccine will die.	**Expressions that sustain inconsistency and overgeneralisation**: 2) One-sided interpretation, 3) Predicting 5) Unsupported judgement or personal opinion / projection”	1. Technically speaking, 100% of the people who get the vaccine will die. You know, eventually. 2. Vaccines should definitely be illegal, think about it: 100% of the people who get them will die. It’s baffling to me that no one talks about this, they are murdering us like it’s nothing!	1. That’s...... actually a good point. But it’s a preventative measure as opposed to a cure as far as I understand it. 2. What does 95% effective mean? That there’s a 5% chance that it won’t work?	1. “There’s nothing wrong with twitter, just follow normal people. I’ve learned more about covid on twitter than I have on here.” 2. Picky, picky: You have a 5% chance of the vaccine not providing any protection from covid, which isn’t the same as a 5% chance of getting sick.
Reddit (Both)	LOL - They don’t even bother to make it believable - New vaccine has been found. 90% effective. DOW jumps 1200 points. Covid is cured!	**1. Expressions that sustain hostility**: 1) Blame, 2) Suspicion **2. Expressions that sustain inconsistency and overgeneralisation such as**: 1) Inflexible unfounded over-generalization, 2) One-sided interpretation, 5) Unsupported judgement or personal opinion / projection”	1. They say the vaccine is 90% effective because the other 10% is actually the Microchip made by Microsoft. 2. The virus is real but the media and people who run the world used it to take down trump. They pumped up death numbers, suppressed the economy, and held back vaccines. They wanted all out misery so people blamed it all on Trump to vote him out.	1. “At this point, i’m willing to take any Vax as long as it means i am free to go out again. I held up ok during the first lockdown then again i was injured and waiting for surgery so i was spending my days under painkillers but the second one is literally killing me! In addition to the bluest of balls i basically regained ability to walk 3 weeks before lockdown 2 soooo Yeay, beside two weeks i spent 2020 locked inside fml, i’m really getting on edge right now...” 2. I mean, in Ireland we’ve been getting updates on this pfizer vaccine nearly weekly. Not everything revolves around America. Just because it’s fresh news for you guys doesn’t mean shit. It’s not a “new vaccine has been found” it’s a vaccine produced by a top drug company that has been under trials for months has passed a phase of trialing.	1. The vaccine or what are you talking about? 2. What is the “risk” to this specific vaccine? The one you have figured it ahead of the people testing it?

## Appendix B: Features of the model: completed list of 98 features

**Table:**

		*Z*-score: Covid vaccine			*Z*-score: disproving conspiracy		
Type	Features	Stigma	Not stigma	Undefined	Stigma	Not stigma	Undefined
Other features	Engagement	− 0.23	− 0.28	− 1.17	− 1.88	0.93	0.31
Other features	Engagement clusters	− 0.06	0.64	− 2.23	− 2.35	1.66	− 1.55
Other features	No. characters	20.12	− 8.71	− 9.06	26.18	− 11.9	− 8.32
Other features	Sentiment score (polarity)	− 7.40	4.03	0.28	− 9.12	4.88	0.08
Other features	Subjectivity score	3.36	− 0.54	− 4.90	1.10	0.23	− 3.24
Summary dimensions	Word count	21.50	− 9.43	− 9.20	26.89	− 12.4	− 8.16
Summary dimensions	Analytical thinking	− 3.20	2.24	− 1.71	− 8.99	8.21	− 13.3
Summary dimensions	Clout: power	− 0.66	0.36	0.03	0.36	− 0.78	2.33
Summary dimensions	Authentic	− 2.19	1.56	− 1.28	− 1.11	1.61	− 3.97
Summary dimensions	Emotional tone	− 5.96	3.54	− 0.84	− 12.8	6.91	− 0.06
Summary dimensions	Words per sentence	7.20	− 1.38	− 9.63	15.49	− 5.46	− 11.3
Summary dimensions	Words > 6 letters	− 5.12	3.83	− 3.64	− 13.4	8.60	− 5.62
Summary dimensions	% words (dictionary)	7.52	− 5.14	3.56	16.93	− 12.0	11.40
Linguistic processes	Function words	10.17	− 4.48	− 4.28	16.65	− 11.1	8.55
Linguistic processes	Pronoun	2.51	− 1.60	0.76	8.47	− 7.87	13.02
Linguistic processes	Ppron (them, itself)	0.46	− 0.66	1.48	4.87	− 5.35	10.73
Linguistic processes	I	− 1.49	0.32	1.88	− 1.30	0.95	− 0.99
Linguistic processes	We	2.82	− 1.29	− 1.02	5.46	− 3.37	1.71
Linguistic processes	You	− 0.80	0.05	1.46	2.10	− 2.56	5.62
Linguistic processes	SheHe	− 1.37	0.26	1.86	− 1.06	− 3.29	15.19
Linguistic processes	They	4.75	− 1.76	− 3.22	9.80	− 4.88	− 1.54
Linguistic processes	Ipron (me, my)	3.07	− 1.57	− 0.50	6.65	− 5.24	6.54
Linguistic processes	Article	7.97	− 3.10	4.87	6.31	− 1.64	− 6.88
Linguistic processes	Prepositions (with, above)	2.70	− 0.50	− 3.67	3.27	− 0.15	− 6.34
Linguistic processes	Auxverb (may, must)	4.78	− 2.56	− 0.35	12.51	− 9.24	9.91
Linguistic processes	Adverbs (really, qucikly)	1.11	− 0.45	− 0.59	− 1.21	− 0.40	4.15
Linguistic processes	Conjunctions (but, whereas)	5.19	− 2.01	− 3.20	2.25	2.25	2.25
Linguistic processes	Negate (not, never)	1.26	− 1.69	3.67	7.39	− 3.23	− 2.91
Other grammar	Verbs	3.66	− 2.68	2.38	5.13	− 4.17	5.56
Other grammar	Adjectives (free, long)	− 1.90	− 0.27	4.87	5.14	− 3.35	2.30
Other grammar	Comparisons (greater, best)	− 1.16	0.56	0.31	− 2.74	1.78	− 1.19
Other grammar	Interrogatives (how, when)	0.47	− 0.35	0.33	− 1.04	− 1.21	6.96
Other grammar	Number	− 1.08	0.43	0.62	− 4.82	2.51	0.31
Other grammar	Quantifiers (few, many)	− 0.42	− 0.49	2.65	3.91	− 1.71	− 1.55
Affect	Affective processes (ugly, bitter)	− 2.88	− 0.09	6.22	0.90	− 2.58	8.23
Affect	Positive emotions (happy, good)	− 5.29	1.84	4.03	− 13.0	4.72	8.94
Affect	Negative emotions (resent, enemy)	3.25	− 3.16	4.99	15.84	− 9.02	1.98
Affect	Anxiety (afraid, tense)	3.79	− 1.95	− 0.55	4.90	− 2.58	− 0.21
Affect	Anger (rage, hurt)	2.65	− 2.14	2.47	8.83	− 5.56	3.21
Affect	Sadness (grief, cry)	− 2.02	0.72	1.48	1.74	− 0.50	− 1.73
Social	Social processes (talk, friend)	− 1.04	− 0.59	4.32	3.13	− 4.81	12.30
Social	Family	− 1.42	− 0.10	3.28	− 0.64	0.19	0.60
Social	Friend	− 1.18	0.09	2.09	− 3.76	1.38	2.55
Social	Female references (girl, her)	1.68	− 1.48	2.01	− 1.83	− 3.28	16.79
Social	Male references (boy, his)	− 2.79	− 0.21	6.49	− 2.50	− 0.09	5.63
Cognitive processes	Cognitive processes (cause, ought)	1.74	− 0.83	− 0.50	5.30	− 3.51	2.61
Cognitive processes	Insight (know, consider)	− 1.33	1.05	− 1.14	− 4.37	2.25	0.39
Cognitive processes	Causation (because, effect)	2.21	− 0.95	− 1.01	3.55	− 1.45	− 1.81
Cognitive processes	Discrepancy (should, could)	3.03	− 1.39	− 1.08	7.92	− 5.13	3.43
Cognitive processes	Tentative (perhaps, guess)	0.37	− 0.40	0.71	4.75	− 2.56	0.02
Cognitive processes	Certainty (always, never)	0.02	− 0.32	1.16	− 0.09	− 1.57	6.39
Cognitive processes	Differentiation (hasn’t, else)	3.46	− 1.24	− 2.50	7.95	− 4.30	0.10
Perceptual processes	Perceptual processes (touch, listen)	− 4.72	2.30	1.18	− 10.5	5.52	0.38
Perceptual processes	Seeing (view, look)	− 3.63	1.40	2.24	− 7.81	3.79	1.62
Perceptual processes	Hearing (listen, sound)	− 2.37	1.19	0.46	− 5.97	3.22	− 0.02
Perceptual processes	Feeling (hold, felt)	− 1.21	0.82	− 0.57	− 2.48	1.92	− 2.30
Biological processes	Biological Processes (eat, blood)	3.30	− 1.63	− 0.74	− 0.15	0.25	− 0.67
Biological processes	Body (heart, cough)	− 0.13	0.16	− 0.33	− 1.86	0.77	0.88
Biological processes	Health (clinic, flu)	4.31	− 2.68	1.077	3.02	− 1.90	1.10
Biological processes	Sexuality (love, incest)	1.88	− 0.67	− 1.39	1.77	− 0.95	− 0.01
Biological processes	Ingestion (swallow, taste)	− 0.99	0.77	− 0.80	− 2.87	1.71	− 0.66
Drives	Drives	0.81	− 2.01	5.74	4.97	− 2.98	1.21
Drives	Affiliation (ally, friend)	− 1.51	− 0.19	3.77	− 0.97	0.96	− 1.71
Drives	Achievement (win, success)	− 1.27	1.01	− 1.11	− 2.09	1.23	− 0.42
Drives	Power (superior, bully)	− 0.23	− 0.19	1.17	2.74	− 1.64	0.65
Drives	Reward (prize, benefit)	1.26	− 2.02	4.85	2.73	− 2.39	3.621
Drives	Risk (danger, doubt)	2.64	− 2.52	3.88	6.79	− 3.34	− 1.28
Time orientation	Focuspast (ago, did)	− 1.95	0.68	1.48	− 5.37	2.49	1.57
Time orientation	Focuspresent (today, now)	5.56	− 3.37	1.06	11.22	− 9.53	13.77
Time orientation	Focusfuture (will, soon)	2.86	− 1.31	− 1.02	8.65	− 5.02	1.47
Relativity	Relativity (exit, area)	− 0.63	1.35	− 3.66	0.30	1.04	− 4.72
Relativity	Motion (walk, move)	0.22	1.12	− 4.59	2.42	0.39	− 6.66
Relativity	Space (down, in)	− 1.89	1.70	− 2.40	1.04	0.001	− 2.19
Relativity	Time (hour, day)	1.66	− 0.69	− 0.85	− 1.62	1.34	− 1.83
Personal concerns	Work (work, boss)	− 2.77	2.16	− 2.29	− 5.07	3.35	− 2.46
Personal concerns	Leisure (house, music)	− 3.04	1.91	− 0.81	− 3.20	2.23	− 2.00
Personal concerns	Home (house, kitchen)	1.54	− 1.09	0.85	0.34	− 0.43	0.97
Personal concerns	Money (audit, cash)	2.81	− 1.11	− 1.66	4.17	− 1.68	− 2.31
Personal concerns	Religion (altar, church)	− 1.93	− 2.10	11.68	2.34	− 2.64	5.43
Personal concerns	Death (bury, kill)	2.95	− 1.20	− 1.63	1.80	− 2.17	4.71
Filler words	Informal	− 3.45	1.24	2.50	− 1.78	0.40	2.19
Filler words	Swear words (damn, shit)	2.60	− 1.91	1.70	10.17	− 5.46	− 0.03
Filler words	Netspeak (lol, thx)	− 3.23	1.48	1.18	0.30	0.10	− 1.03
Filler words	Assent (agree, yes)	− 2.39	1.45	− 0.44	− 4.63	2.97	− 1.89
Filler words	Nonfluencies (uh, rr*)	− 2.36	0.81	1.85	− 2.92	− 0.06	6.41
Filler words	Fillers (blah, you know)	− 0.49	− 0.99	4.64	3.49	− 2.27	1.54
Punctuation marks	AllPunc	− 5.14	2.12	2.69	0.63	− 0.49	0.58
Punctuation marks	Period	− 3.45	1.21	2.60	0.71	− 0.46	0.32
Punctuation marks	Comma	− 0.87	1.00	− 1.91	− 1.47	1.33	− 2.13
Punctuation marks	Colon	− 1.02	0.91	− 1.29	− 4.46	3.28	− 3.47
Punctuation marks	SemiC	1.31	− 0.95	0.82	0.46	− 0.06	− 0.75
Punctuation marks	QMark	− 1.51	0.45	1.43	− 0.65	0.03	1.25
Punctuation marks	Exclam	− 2.30	0.61	2.44	1.00	− 0.61	0.31
Punctuation marks	Dash	− 2.22	1.80	− 2.10	− 1.08	1.31	− 2.87
Punctuation marks	Quote	1.15	− 0.41	− 0.85	− 0.55	0.57	− 1.09
Punctuation marks	Apostro	− 0.99	− 0.30	3.13	2.45	− 2.41	4.32
Punctuation marks	Parenth	− 0.31	1.01	− 3.08	− 0.23	− 0.27	1.54
Punctuation marks	OtherP	− 2.72	2.06	− 2.01	− 2.33	1.44	− 0.72

## Abbreviations

CNN: Convolutional neural network

## References

[1] Katz I (2014). Stigma: a social psychological analysis, 3rd edn., pp 1–32.

[2] Joseph AJ, Tandon N, Yang LH, Duckworth K, Torous J, Seidman LJ, Keshavan MS (2015). Schizophrenia: use and misuse on Twitter. Schizophr Res.

[3] Merriam-Webster. https://www.merriam-webster.com/dictionary/stigma. Accessed 28 Mar 2021

[4] Goffman E (1986). Stigma: notes on the management of spoiled identity. A Touchstone book, pp 1–168.

[5] Allport GW (1958). The nature of prejudice, 2nd edn., pp 1–526.

[6] Glick P, Fiske ST (1996). The ambivalent sexism inventory: differentiating hostile and benevolent sexism. J Pers Soc Psychol.

[7] Fiske ST, Xu J, Cuddy AC, Glick P (1999). (Dis) respecting versus (dis) liking: status and interdependence predict ambivalent stereotypes of competence and warmth. J Soc Issues.

[8] Freedman JL, Sears DO (1965) Selective exposure. Adv Exp Soc Psychol (2):57–97 10.1037/h0022380 14333316

[9] Paluck EL, Green SA, Green DP (2019). The contact hypothesis re-evaluated. Behav Public Policy.

[10] Nickerson RS (1998). Confirmation bias: a ubiquitous phenomenon in many guises. Rev Gen Psychol.

[11] Klayman J (1995). Varieties of confirmation bias. Psychol Learn Motiv.

[12] Oswald ME, Grosjean S (2004) Confirmation bias. Cognitive illusions: a handbook on fallacies and biases in thinking, judgement and memory, vol 79

[13] Festinger L (1957). A theory of cognitive dissonance.

[14] Straton N, Jang H, Ng R, Vatrapu R, Mukkamala RR (2019) Computational modeling of stigmatised behaviour in pro-vaccination and anti-vaccination discussions on social media. In: 2019 IEEE international conference on bioinformatics and biomedicine (BIBM), vol 11. IEEE, pp 2673–2681

[15] Stephan WG, Stephan CW (1985). Intergroup anxiety. J Soc Issues.

[16] CBSnews. Available online: 24 March 2021. https://www.cbsnews.com/news/covid-vaccine-disinformation-twitter-facebook-state-attorneys-general/https://www.cbsnews.com/news/covid-vaccine-disinformation-twitter-facebook-state-attorneys-general/https://www.cbsnews.com/news/covid-vaccine-disinformation-twitter-facebook-state-attorneys-general/ . Accessed 25 Sep 2021

[17] CBSnews. Available online: 25 March 2021. https://www.cbsnews.com/news/vaccine-disinformation-social-media-center-for-countering-digital-hate-report/https://www.cbsnews.com/news/vaccine-disinformation-social-media-center-for-countering-digital-hate-report/https://www.cbsnews.com/news/vaccine-disinformation-social-media-center-for-countering-digital-hate-report/. Accessed 25 Sep 2021

[18] BBC. Available online: 10 August 2021. https://www.bbc.com/news/blogs-trending-58167339. Accessed 25 Sep 2021

[19] First Draft News. Available online: 17 March 2021. https://firstdraftnews.org/articles/rt-fringe-undermine-covid-vaccinations/. Accessed 25 Sep 2021

[20] First Draft News. Available online: 24 March 2021. https://firstdraftnews.org/articles/vaccine-infertility-claims-youtube-fringe/. Accessed 25 Sep 2021

[21] Johnson NF, Velásquez N, Restrepo NJ, Leahy R, Gabriel N, El Oud S, Lupu Y (2020). The online competition between pro-and anti-vaccination views. Nature.

[22] NBCnews. Available online: 20 November 2020. https://www.nbcnews.com/tech/tech-news/covid-19-vaccines-face-varied-powerful-misinformation-movement-online-n1249378. Accessed 25 Sep 2021

[23] SFchronicle. Available online: 8 March 2021. https://www.sfchronicle.com/opinion/openforum/article/What-Facebook-is-doing-to-combat-vaccine-hesitancy-16007494.php. Accessed 25 Sep 2021

[24] Straton N, Ng R, Jang H, Vatrapu R, Mukkamala RR (2019) Predictive modelling of stigmatised behaviour in vaccination discussions on Facebook. In: 2019 IEEE international conference on bioinformatics and biomedicine (BIBM), vol 11. IEEE, pp 2561–2568

[25] First Draft News. Available online: 6 May 2021. https://firstdraftnews.org/articles/vaccine-misinformation-in-facebook-comment-sections-a-case-study/. Accessed 25 Sep 2021

[26] Reavley NJ, Pilkington PD (2014). Use of Twitter to monitor attitudes toward depression and schizophrenia: an exploratory study. PeerJ.

[27] Li A, Jiao D, Zhu T (2018). Detecting depression stigma on social media: a linguistic analysis. J Affect Disord.

[28] Li A, Jiao D, Liu X, Zhu T (2020). A comparison of the psycholinguistic styles of schizophrenia-related stigma and depression-related stigma on social media: content analysis. J Med Internet Res.

[29] Li A, Jiao D, Liu X, Zhu T (2018). An analysis of stigma and suicide literacy in responses to suicides broadcast on social media. Asia Pac Psychiatry.

[30] Reich JA (2018) “We are fierce, independent thinkers and intelligent”: social capital and stigma management among mothers who refuse vaccines. Soc Sci Med:112015 10.1016/j.socscimed.2018.10.027 30442504

[31] Robinson P, Turk D, Jilka S, Cella M (2019). Measuring attitudes towards mental health using social media: investigating stigma and trivialisation. Soc Psychiatry Psychiatr Epidemiol.

[32] Oscar N, Fox PA, Croucher R, Wernick R, Keune J, Hooker K (2017). Machine learning, sentiment analysis, and tweets: an examination of Alzheimer’s disease stigma on Twitter. J Gerontol Ser B Psychol Sci Soc Sci.

[33] Lydecker JA, Cotter EW, Palmberg AA, Simpson C, Kwitowski M, White K, Mazzeo SE (2016). Does this Tweet make me look fat? a content analysis of weight stigma on Twitter. Eat Weight Disord-Studies on Anorexia, Bulimia and Obesity.

[34] Hussin M, Frazier S, Thompson JK (2011). Fat stigmatisation on YouTube: a content analysis. Body Image.

[35] Moore D, Ayers S, Drey N (2016). A thematic analysis of stigma and disclosure for perinatal depression on an online forum. JMIR Mental Health.

[36] Budenz A, Klassen A, Purtle J, Yom Tov E, Yudell M, Massey P (2020). Mental illness and bipolar disorder on Twitter: implications for stigma and social support. Int J Ment Health.

[37] Budhwani H, Sun R (2020). Creating COVID-19 stigma by referencing the novel Coronavirus as the “Chinese virus” on Twitter: quantitative analysis of social media data. J Med Internet Res.

[38] Burki T (2020). The online anti-vaccine movement in the age of COVID-19. The Lancet Digital Health.

[39] Fridman A, Gershon R, Gneezy A (2021). COVID-19 and vaccine hesitancy: a longitudinal study. PloS one.

[40] Johnson NF, Velasquez N, Restrepo NJ, Leahy R, Gabriel N, El Oud Sara, Zheng M, Manrique P, Wuchty S, Lupu Y (2020). The online competition between pro-and anti-vaccination views. Nature.

[41] Puri N, Coomes EA, Haghbayan H, Gunaratne K (2020). Social media and vaccine hesitancy: new updates for the era of COVID-19 and globalized infectious diseases. Human Vaccines & Immunotherapeutics.

[42] BBC. Available online: 8 December 2020. https://www.bbc.com/news/uk-55227325. Accessed 01 Mar 2021

[43] Octoparse. https://www.octoparse.com/#. Accessed 04 Feb 2021

[44] YouTube Scrapper. https://ytcommentscraper.getwebooster.com. Accessed 10 Feb 2021

[45] Fiske ST (2000). Stereotyping prejudice, and discrimination at the seam between the centuries: evolution, culture, mind, and brain. Eur J Soc Psychol.

[46] Link BG, Phelan JC (2020). Conceptualizing stigma. Annu Rev Sociol.

[47] Fiske ST (1998). Stereotyping, prejudice and discrimination.

[48] Straton N, Jang H, Ng R (2020) Stigma annotation scheme and stigmatised language detection in Health-Care discussions on social media. In: Proceedings of the 12th language resources and evaluation conference, vol 05. pp 1178–1190

[49] Artstein R, Poesio M (2008). Inter-coder agreement for computational linguistics. Comput Linguist.

[50] Fleiss JL (1971). Measuring nominal scale agreement among many raters. Psychol Bull.

[51] LIWC. https://www.liwc.app. Accessed 28 Mar 2021

[52] www.statisticshowto.com. Available online: 2021 https://www.statisticshowto.com/probability-and-statistics/hypothesis-testing/f-test/. Accessed 25 Sep 2021

[53] Robert N, Gary M, Yale K (2018) Model evaluation and enhancement. Handbook of statistical analysis and data mining applications, pp 215–233

[54] techcrunch.com. Available online: 30 Oct 2018. https://techcrunch.com/2018/10/30/twitters-doubling-of-character-count-from-140-to-280-had-little-impact-on-length-of-tweets/ . Accessed 25 Sep 2021

[55] techpostplus.com. Available online: 29 Jun 2020. https://techpostplus.com/youtube-video-comment-faqs/. Accessed 25 Sep 2021

[56] support.google.com. Available online: 16 Nov 2019. https://support.google.com/youtube/thread/20057621/why-does-youtube-comment-s-replies-have-a-max-of-501?hl=en. Accessed 25 Sep 2021

[57] Wikipedia 2014 + Gigaword 5, 6B tokens, 400K vocab, uncased, 50d, vectors, 822 MB download. https://nlp.stanford.edu/projects/glove/. Accessed 25 Sep 2021

[58] Common Crawl , 42B tokens, 1.9M vocab, uncased, 300d vectors, 1.75 GB download. https://nlp.stanford.edu/projects/glove/. Accessed 25 Sep 2021

[59] Common Crawl (840B tokens, 2.2M vocab, cased, 300d vectors, 2.03 GB download). https://nlp.stanford.edu/projects/glove. Accessed 25 Sep 2021

[60] 1 million word vectors trained on Wikipedia 2017, UMBC webbase corpus and statmt.org news dataset (16B tokens). https://fasttext.cc/docs/en/crawl-vectors.html. Accessed 25 Sep 2021

[61] 2 million word vectors trained on Common Crawl (600B tokens). https://fasttext.cc/docs/en/crawl-vectors.html. Accessed 25 Sep 2021

[62] Vector size 1024, English Wikipedia Dump of October 2019, Lemmatization. http://vectors.nlpl.eu/repository/20/209.zip. Accessed 25 Sep 2021

[63] Vector size 300, Window size 3, English Wikipedia Dump of October 2019, Lemmatization. http://vectors.nlpl.eu/repository/20/200.zip. Accessed 25 Sep 2021

[64] Weintraub W (2003) Verbal behavior and personality assessment. The Psychological Assessment of Political Leaders with Profiles of Saddam Hussein and Bill Clinton:137–152

[65] Tausczik YR, Pennebaker JW (2010) The psychological meaning of words: LIWC and computerized text analysis methods. J Lang Soc Psychol:24–54

[66] Pennebaker JW, Mayne TJ, Francis ME (1997). Linguistic predictors of adaptive bereavement. J Personal Soc Psychol.

[67] Pennebaker JW, Boyd RL, Jordan K, Blackburn K (2015) The development and psychometric properties of LIWC2015

[68] Holtzman NS, Tackman AM, Carey AL, Brucks MS, Küfner ACP, Deters FG, Back MD, Donnellan MB, Pennebaker JW, Sherman RA (2019). Linguistic markers of grandiose narcissism: a LIWC analysis of 15 samples. J Lang Soc Psychol.

[69] del Pilar Salas-Zárate M, López-López E, Valencia-García R, Aussenac-Gilles N, Almela Á, Alor-Hernández G (2014). A study on LIWC categories for opinion mining in Spanish reviews. J Inf Sci.

[70] Robinson RL, Navea R, Ickes W (2013). Predicting final course performance from students’ written self-introductions: a LIWC analysis. J Lang Soc Psychol.

[71] Higuchi K (2016). KH Coder 3 reference, manual.

[72] Allport G (1954). The nature of prejudice.

[73] Aronson E (1978). The jigsaw classroom.

[74] Hewstone ME, Brown RE (1986). Contact and conflict in intergroup encounters.

[75] Pettigrew TF, Tropp LR (2006). A meta-analytic test of intergroup contact theory. J Pers Soc Psychol.

[76] Pettigrew TF (1998). Intergroup contact theory. Annu Rev Psychol.

[77] Pettigrew TF, Tropp LR, Wagner U, Christ O (2011). Recent advances in intergroup contact theory. Int J Intercult Relat.

[78] Sherif M (1975) On the application of superordinate goals theory. Soc Sci Q JSTOR:510–512

[79] McClendon MJ (1974). Interracial contact and the reduction of prejudice. Sociol Focus.

[80] Amir Y (1969). Contact hypothesis in ethnic relations. Psychol Bull.

[81] Rothbart M, John OP (1985). Social categorization and behavioral episodes: a cognitive analysis of the effects of intergroup contact. J Soc Issues.

